# *FOXM1* repression increases mitotic death upon antimitotic chemotherapy through *BMF* upregulation

**DOI:** 10.1038/s41419-021-03822-5

**Published:** 2021-05-25

**Authors:** Sara Vaz, Fábio J. Ferreira, Joana C. Macedo, Gil Leor, Uri Ben-David, José Bessa, Elsa Logarinho

**Affiliations:** 1 i3S - Instituto de Investigação e Inovação em Saúde, Universidade do Porto, 4200-135 Porto, Portugal; 2 Aging and Aneuploidy Group, IBMC - Instituto de Biologia Molecular e Celular, Universidade do Porto, 4200-135 Porto, Portugal; 3 Programa doutoral em Biologia Molecular e Celular, Instituto de Ciências Biomédicas Abel Salazar, Universidade do Porto, 4050-313 Porto, Portugal; 4 Vertebrate Development and Regeneration Group, IBMC - Instituto de Biologia Molecular e Celular, Universidade do Porto, 4200-135 Porto, Portugal; 5 Graduate Program in Areas of Basic and Applied Biology (GABBA), Instituto de Ciências Biomédicas Abel Salazar (ICBAS), Universidade do Porto, 4050-313 Porto, Portugal; 6 Department of Human Molecular Genetics & Biochemistry, Faculty of Medicine, Tel Aviv University, Tel Aviv, Israel

**Keywords:** Chemotherapy, Mitosis, Transcriptional regulatory elements

## Abstract

Inhibition of spindle microtubule (MT) dynamics has been effectively used in cancer treatment. Although the mechanisms by which MT poisons elicit mitotic arrest are fairly understood, efforts are still needed towards elucidating how cancer cells respond to antimitotic drugs owing to cytotoxicity and resistance side effects. Here, we identified the critical G2/M transcription factor Forkhead box M1 (FOXM1) as a molecular determinant of cell response to antimitotics. We found FOXM1 repression to increase death in mitosis (DiM) due to upregulation of the BCL-2 modifying factor (*BMF*) gene involved in anoikis, an apoptotic process induced upon cell detachment from the extracellular matrix. FOXM1 binds to a *BMF* intronic cis-regulatory element that interacts with both the *BMF* and the neighbor gene *BUB1B* promoter regions, to oppositely regulate their expression. This mechanism ensures that cells treated with antimitotics repress *BMF* and avoid DiM when FOXM1 levels are high. In addition, we show that this mechanism is partly disrupted in anoikis/antimitotics-resistant tumor cells, with resistance correlating with lower *BMF* expression but in a FOXM1-independent manner. These findings provide a stratification biomarker for antimitotic chemotherapy response.

## Introduction

Drugs interfering with spindle microtubule (MT) dynamics, MT poisons, have been effectively used as chemotherapeutic agents targeting cancer cell proliferation^1^. Notwithstanding substantial research elucidating their mechanism of action, how the clinical response is achieved remains elusive. MT poisons, also known as antimitotic drugs, inhibit the metaphase-to-anaphase transition by activating the spindle assembly checkpoint due to the production of defective chromosome attachments to the spindle^2^. Yet, cancer cells were found to display profound intra- and inter-line variation on their response to antimitotic drugs. This variability is determined by two competing networks: one that protects Cyclin B1 from degradation and another that leads to caspase activation^3^. If Cyclin B1 levels drop below the threshold needed to maintain Cdk1 activity during prolonged exposure to antimitotics, the cell exits mitosis as a polyploid entity (slippage). If apoptotic signals accumulate faster than Cyclin B1 degradation, the cell dies in mitosis (death in mitosis, DiM). Consequently, understanding the cross-talk between these two pathways is critical to foresee and modulate chemotherapy efficacy.

The Forkhead Box M1 (FOXM1) transcription factor primarily regulates the proliferation-associated gene cluster critical to G2/M transition, mitotic spindle assembly, and chromosome segregation^4,5^. Aberrant *FOXM1* overexpression is found in most human cancers and is a major adverse prognostic marker^6,7^. Moreover, *FOXM1* upregulation has been implicated in the development of chemotherapeutic resistance, viz. to antimitotic paclitaxel^8–14^, even though the molecular mechanisms remain largely unknown.

Resistance to paclitaxel has been associated with evasion to anoikis^15–18^, an apoptotic process that adherent cells normally undergo upon loss of contact with the extracellular matrix or neighboring cells^19^. Cancer cells evade this process to survive after detachment from the primary sites and be able to metastasize^20^. Anoikis execution has been linked to the BH3-only pro-apoptotic proteins of the mitochondrial (intrinsic) apoptotic pathway. In particular, BMF (BCL-2-modifying factor) and BIM (*BCL2L11*, BCL-2 like 11), which counteract the anti-apoptotic activity of BCL-2, BCL-xL, and MCL-1^19,21–23^, or directly activate the pore-former proteins at the mitochondria^24^. In addition, BMF interacts with the dynein light chain DYNLL1 and DYNLL2 isoforms^25^, suggesting a putative role in cytoskeleton stress sensing^26^.

Here, we investigated whether *FOXM1* expression levels determine cell fate decision upon antimitotic drug treatment. Using time-lapse live-cell imaging to monitor individual mitotic cells, we found that *FOXM1* repression leads to increased DiM due to upregulation of the pro-apoptotic *BMF* gene. FOXM1 binding to a *BMF* intronic cis-regulatory element (CREs) acts to repress *BMF* expression while enhancing the expression of the *BUB1B* neighbor gene. Through this essential mechanism, mitotic cells are able to circumvent anoikis induction during prolonged arrest unless *FOXM1* is repressed. However, this mechanism is partially disrupted in anoikis-resistant tumor cells. Resistance to anoikis-inducing drugs, including paclitaxel, correlates with lower levels of *BMF* in a FOXM1-independent manner. This provides a useful biomarker for stratification of tumor response to antimitotic chemotherapy.

## Results

### FOXM1 expression modulates cell fate profile in response to antimitotics

**Fig. 1:**
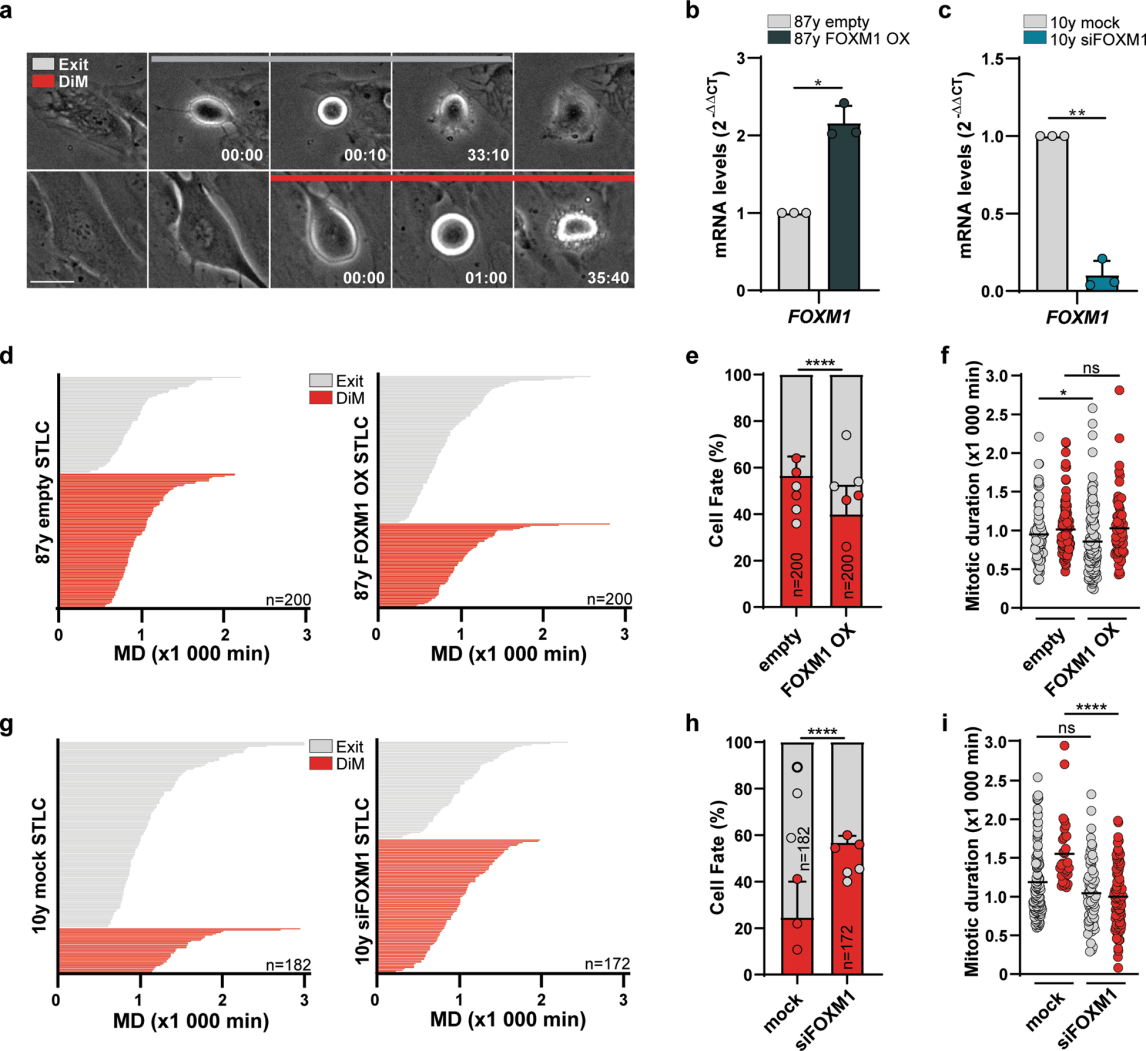
FOXM1 levels modulate cell fate profile in response to antimitotics. **a** Representative time-lapse sequences of slippage/mitotic exit (in gray) and death in mitosis (DiM, in red) cell fates of HDFs treated with antimitotics. Time in h:min, from nuclear envelope breakdown (NEB) to each cellular outcome. Scale bar, 20 µm. **b** *FOXM1* transcript levels in 87-year-old HDFs transduced with control (empty vector) and FOXM1-overexpressing lentiviruses. **c** *FOXM1* transcript levels in mock- and FOXM1 siRNA-depleted 10-year-old HDFs. **d** Individual cell fate profiling (exit vs. DiM) of control (empty) (*n* = 200) or FOXM1-overexpressing 87 y HDFs (*n* = 200) treated with 5 µM STLC. MD, mitotic duration. **e** Cell fate percentage of the experiments described in **d**. **f** Mitotic duration of the individual cell fates shown in **d**. **g** Individual cell fate profiling (exit vs. DiM) of mock- (*n* = 182) and siFOXM1-depleted (*n* = 172) 10 y HDFs treated with 5 µM STLC. MD, mitotic duration. **h** Cell fate percentage of the experiments described in **g**. **i** Mitotic duration of the individual cell fates shown in **g**. Data information: In **b**, **c** data are mean ± S.D. from *n* = 3 independent experiments; *p ≤ 0.05, **p ≤ 0.01 (two-tailed paired *t* test). In **e**, **h** data are mean ± S.D. from *n* = 3 independent experiments; ****p ≤ 0.0001 (two-tailed *χ*^2^). In **f**, **i** values are mean. *p ≤ 0.05, ****p ≤ 0.0001 (two-tailed Mann–Whitney test).

We used time-lapse imaging of human dermal fibroblasts (HDFs) (young vs. aged) with distinct FOXM1 levels (high vs. low)^27^ to investigate individual cell response (slippage/exit vs. DiM) to antimitotic drugs (Fig. 1a). To ensure maximal mitotic blockage and apoptosis response, we treated cells with saturating concentrations of different antimitotic drugs, namely *S*-trityl-l-cysteine (STLC) that inhibits kinesin-5/Eg5, and the classic MT poison paclitaxel (TX). In both, 87-year-old HDFs (low FOXM1) were lentiviral-transduced to express a constitutively active form of FOXM1 (FOXM1 OX) (Fig. 1b; Fig. S1a), whereas 10-year-old HDFs (high FOXM1) were transfected with FOXM1 siRNA (siFOXM1) (Fig. 1c; Fig. S1b). We found that FOXM1 induction in elderly cells shifted their cell fate profile towards mitotic exit in both STLC (Fig. 1d, e) and TX treatments (Fig. S1c, d). Conversely, siRNA-mediated FOXM1 repression in young fibroblasts shifted their cell fate profile towards DiM in comparison with controls (Fig. 1g, h; Fig. S1f, g), which is in agreement with increased DiM also found in aged vs. young HDFs. The siRNA-driven shift towards DiM was not due to any differences in antimitotic drug action (Fig. S1i), and was specifically due to FOXM1 repression, as evaluated by FOXM1 OX rescue experiment (Fig. S1j, k). We additionally measured the time cells took to execute exit or DiM in response to STLC and TX. We found FOXM1 induction to accelerate slippage and FOXM1 repression to accelerate DiM (Fig. 1f, i; Fig. S1e, h), thus suggesting that FOXM1 modulates both competing networks. Moreover, we assessed the degradation rate of GFP-tagged Cyclin B1 in young and elderly cells (Fig. S2a, b). Despite the lower Cyclin B1 levels in elderly cells as previously reported^27^, we observed that cyclin B1 is degraded slower in elderly cells (Fig. S2b) thereby explaining why these cells are not more slippage-prone than younger cells. This is possibly owing to downregulation of the Cdc20 co-factor of APC/C activity^27^. In addition, we tested non-RNAi modalities for FOXM1 repression, namely the small-molecule inhibitors RCM-1^28^, which inhibits FOXM1 nuclear localization, and FDI-6^29^, which inhibits FOXM1 binding to DNA. Both inhibitors shifted cell fate profiling of young cells towards increased DiM in response to paclitaxel (Fig. S2c–h), supporting that FOXM1 repression promotes DiM via its canonical role as transcription factor.

### BMF is an effector of DiM driven by FOXM1 inhibition in response to antimitotics

**Fig. 2:**
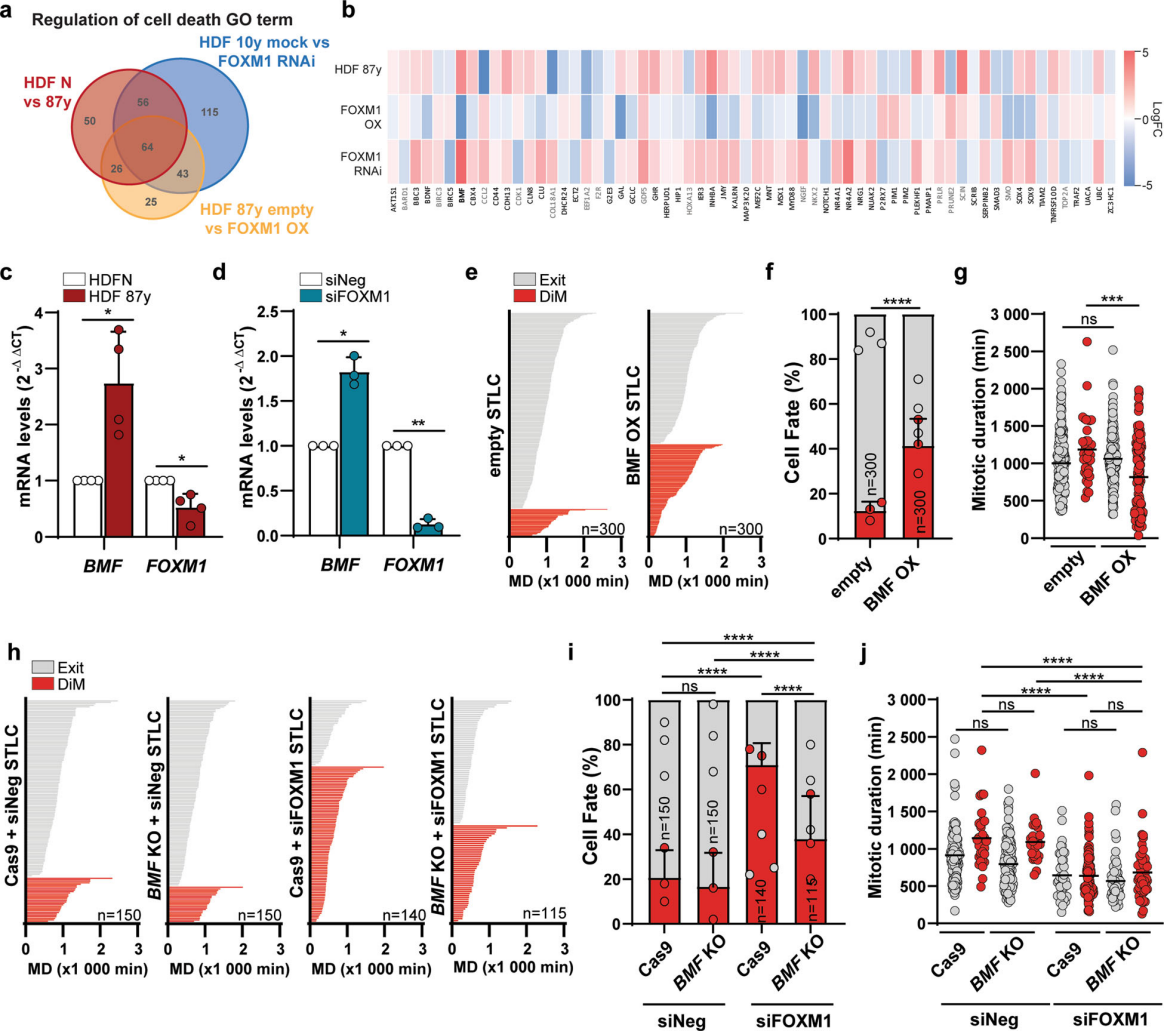
BMF upregulation under low FOXM1 levels accounts for the shift towards DiM. **a** Venn diagram of the cell death GO term genes (DAVID Functional Annotation^52^), found significantly altered in the RNA-seq data sets of N vs. 87 y, 10 y mock vs. FOXM1 RNAi, and 87 y empty vs. FOXM1-overexpressing mitotic HDFs^27^. **b** Heatmap of the 64 overlapping cell death genes shown in **a**. FC, fold change. **c** *BMF* and *FOXM1* transcript levels in mitotic cells from N vs. 87 y HDFs. **d** *BMF* and *FOXM1* transcript levels in mitotic cells from siNeg vs. siFOXM1-depleted neonatal HDFs. **e** Individual cell fate profiling of control (empty vector) (*n* = 300) or BMF-overexpressing neonatal HDFs (*n* = 300) treated with 5 µM STLC. MD, mitotic duration. **f** Cell fate percentage of the experiments described in **e**. **g** Mitotic duration of the individual cell fates shown in **e**. **h** Individual cell fate profiling of CRISPR/Cas9-edited control (Cas9) or BMF (*BMF* KO) HDFs, depleted with Neg or FOXM1 siRNAs, and treated with 5 µM STLC. MD, mitotic duration. *n* = cell number. **i** Cell fate percentage of the experiments described in **h**. **j** Mitotic duration of the individual cell fates shown in **h**. Data information: In **c**, **d** data are mean ± S.D. from *n* = 4 and *n* = 3 independent experiments; *p ≤ 0.05, **p ≤ 0.01 (two-tailed paired *t* test). In **f**, **i** data are mean ± S.D. of *n* = 3 independent experiments; ****p ≤ 0.0001 (two-tailed *χ*^2^). In **g** values are mean; ***p ≤ 0.001 (two-tailed Mann–Whitney test). In **j** values are mean; ****p ≤ 0.0001 (two-tailed Kruskal–Wallis and Dunn’s multiple comparison test).

As FOXM1 inhibition accelerates DiM, this suggests that FOXM1 influences the death pathway of the competing networks model. Thus, we interrogated the “regulation of cell death” GO term in our previous RNA-seq data sets generated from mitotic HDFs blocked with STLC^27^ (Fig. 2a). We found 64 differently altered genes across the neonatal vs. 87 y HDFs, mock vs. siFOXM1 10 y HDFs, and empty vs. FOXM1 OX 87 y HDFs RNA-seq data sets. Forty-eight out of these 64 genes exhibited similar transcriptional changes in elderly and siFOXM1-depleted cells, which were reverted upon FOXM1 overexpression (Fig. 2b). The most downregulated gene in FOXM1 RNAi was *BIRC5*, which encodes the well-established pro-survival factor known as Survivin. The most upregulated gene was *BMF*, which similarly to *BBC3* (PUMA) and *PMAIP1* (NOXA), encodes for a BH3-only pro-apoptotic protein^30^. BMF captured our attention because, like BIM, it is reported as an effector of anoikis^19^, and anoikis has been associated with resistance to paclitaxel^15–18^. Thus, we wondered whether FOXM1 repression in cells under prolonged mitotic arrest shifts their fate towards DiM through *BMF* upregulation and anoikis induction. qPCR analysis confirmed *BMF* upregulation in elderly (87 y) and siFOXM1-depleted young (10 y) cells arrested in mitosis with STLC in comparison with controls (Fig. 2c–d). BMF protein levels could not be monitored owing to lack of available specific antibodies as reported by others^19,31^. To address the functional role of BMF in mitotic cell fate response to antimitotics, neonatal HDFs (HDF N) were lentiviral-transduced to express FLAG-tagged BMF at an efficiency of ~40% (Fig. S3a, b). We found that BMF overexpression significantly increased and accelerated DiM in response to STLC (Fig. 2e–g) and TX (Fig. S3c–e). Conversely, CRISPR/Cas9-mediated knockout of *BMF* (Fig. S3f, g) significantly rescued the shift towards DiM observed in siFOXM1- vs. siNeg-depleted cell cultures treated with STLC and TX (Fig. 2h–i; Fig. S3h–i). However, *BMF* knockout did not alter the time cells took to exit or die in mitosis, suggesting that the cyclin B1 degradation pathway remained unchanged (Fig. 2j; Fig. S3j). In addition, we verified that in the absence of antimitotics, neither *FOXM1* repression nor *BMF* overexpression was able to induce DiM (Fig. S4a–c), thus indicating that a mitotic delay is needed to mount the apoptotic signaling, and that *FOXM1* and *BMF* modulate cell death under priming conditions of prolonged mitotic arrest. Overall, these data show that BMF accounts for increased DiM in response to antimitotic drugs under low FOXM1 transcriptional activity.

### FOXM1 is required to inhibit *BMF* expression during mitosis

**Fig. 3:**
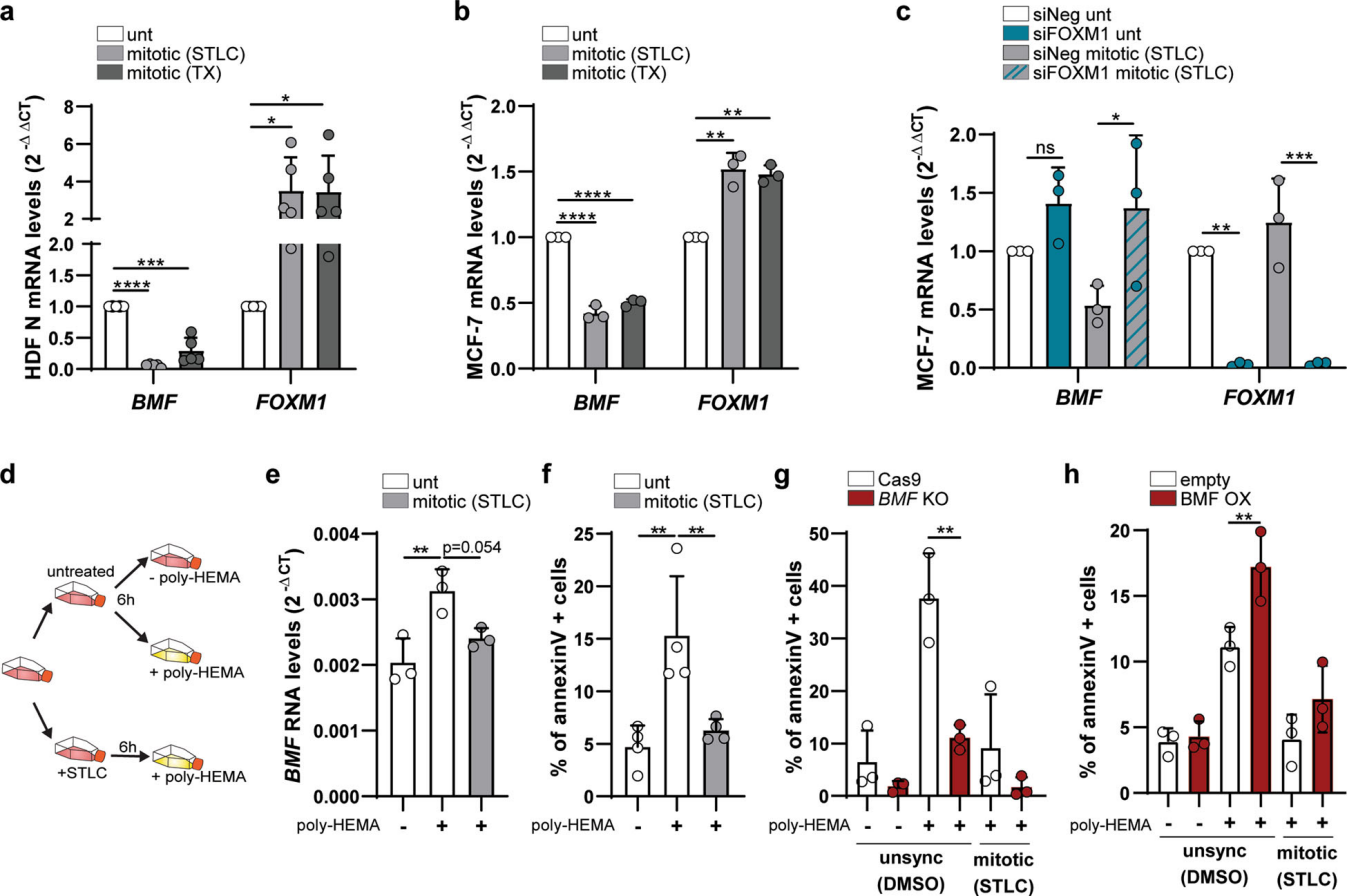
FOXM1 represses *BMF* gene expression during mitosis to prevent anoikis. **a** *BMF* and *FOXM1* transcript levels in asynchronous (untreated, unt) and mitotic neonatal HDFs (shake-off upon STLC or TX treatment). **b** *BMF* and *FOXM1* transcript levels in asynchronous (unt) and mitotic MCF-7 cells (shake-off upon STLC or TX treatment). **c** *BMF* and *FOXM1* transcript levels in asynchronous (unt) and mitotic (shake-off upon STLC) MCF-7 cells depleted with Neg or FOXM1 siRNAs. **d** Experimental layout for the assessment of anoikis sensitivity in asynchronous (untreated) vs. mitotic-enriched (STLC treatment) MCF-7 cells cultured in poly-HEMA-coated flasks. **e** *BMF* transcript levels in the cell culture conditions depicted in **d**. **f** Annexin V/apoptosis cytometry analysis in the cell culture conditions depicted in **d**. **g** Annexin V cytometry analysis in the cell culture conditions depicted in **d**, using CRISPR/Cas9-edited control (Cas9) or BMF (*BMF* KO) MCF-7 cells. **h** Annexin V cytometry analysis in the cell culture conditions depicted in **d**, using MCF-7 cells lentiviral-transduced with empty or pLVX-BMF (BMF OX) plasmids. Data information: in **a**, **b** data are mean ± S.D. from *n* = 5 and *n* = 3 independent experiments; *p ≤ 0.05, **p ≤ 0.01, ***p ≤ 0.001, ****p ≤ 0.0001 (two-tailed one-way ANOVA and Dunnett’s multiple comparison test). In **c**, **e**–**h** data are mean ± S.D. from *n* = 3 or *n* = 4 independent experiments; *p ≤ 0.05, **p ≤ 0.01, ***p ≤ 0.001 (two-tailed one-way ANOVA and Tukey’s multiple comparison test).

As FOXM1 is active at the G2/M cell cycle transition and we found FOXM1 repression to induce *BMF* upregulation, we hypothesized that *BMF* gene expression is cell cycle-regulated. To test this, we performed a mitotic shake-off of neonatal cultures treated with STLC and TX. qPCR analysis of mitotic cell fractions vs. asynchronous cell population revealed pronounced downregulation of *BMF* in arrested mitotic cells expressing high *FOXM1* levels (Fig. 3a). Similar results were found using an alternative cellular model, the MCF-7 breast cancer epithelial cell line (Fig. 3b), in which FOXM1 repression also induces *BMF* upregulation (Fig. 3c). In addition, qPCR analysis of an MCF-7 G2-enriched cell population retrieved 7 h upon thymidine block washout (before apparent mitotic roundup) (Fig. S5a) confirmed that *BMF* is already downregulated in G2 and thus, not merely due to the global transcriptional shutdown during mitosis (Fig. S5b).

Next, we asked whether *BMF* repression during G2/M is critical to prevent anoikis during prolonged mitotic arrest. To test this idea, we plated asynchronous and mitosis-enriched (6 h STLC treatment) MCF-7 cell populations in poly-2-hydroxyethyl methacrylate (poly-HEMA)-coated dishes to measure the extent of anoikis induced by the poly-HEMA inhibition of cell adherence (Fig. 3d). qPCR analysis of *BMF* expression (Fig. 3e) and annexin V cytometry (Fig. 3f) were used as readouts for anoikis after 6 h cell culture in poly-HEMA-coated dishes. We found *BMF* upregulation and increased apoptosis in the MCF-7 asynchronous cell population (mostly G1, see Fig. S5a) but not in the mitotic subpopulation. Apoptosis in the MCF-7 asynchronous population was specifically due to BMF, as evaluated by a rescue experiment using *BMF* KO MCF-7 cells (Fig. 3g). Conversely, *BMF* overexpression in MCF-7 cells turned cells more sensitive to poly-HEMA (Fig. 3h). Thus, we conclude that *BMF* expression is cell cycle-regulated, being repressed during G2/M when FOXM1 is transcriptionally active. Importantly, this regulation precludes prolonged mitotic cell rounding from being perceived as cell detachment and from inducing anoikis.

### FOXM1 acts as a *BMF* transcriptional repressor

**Fig. 4:**
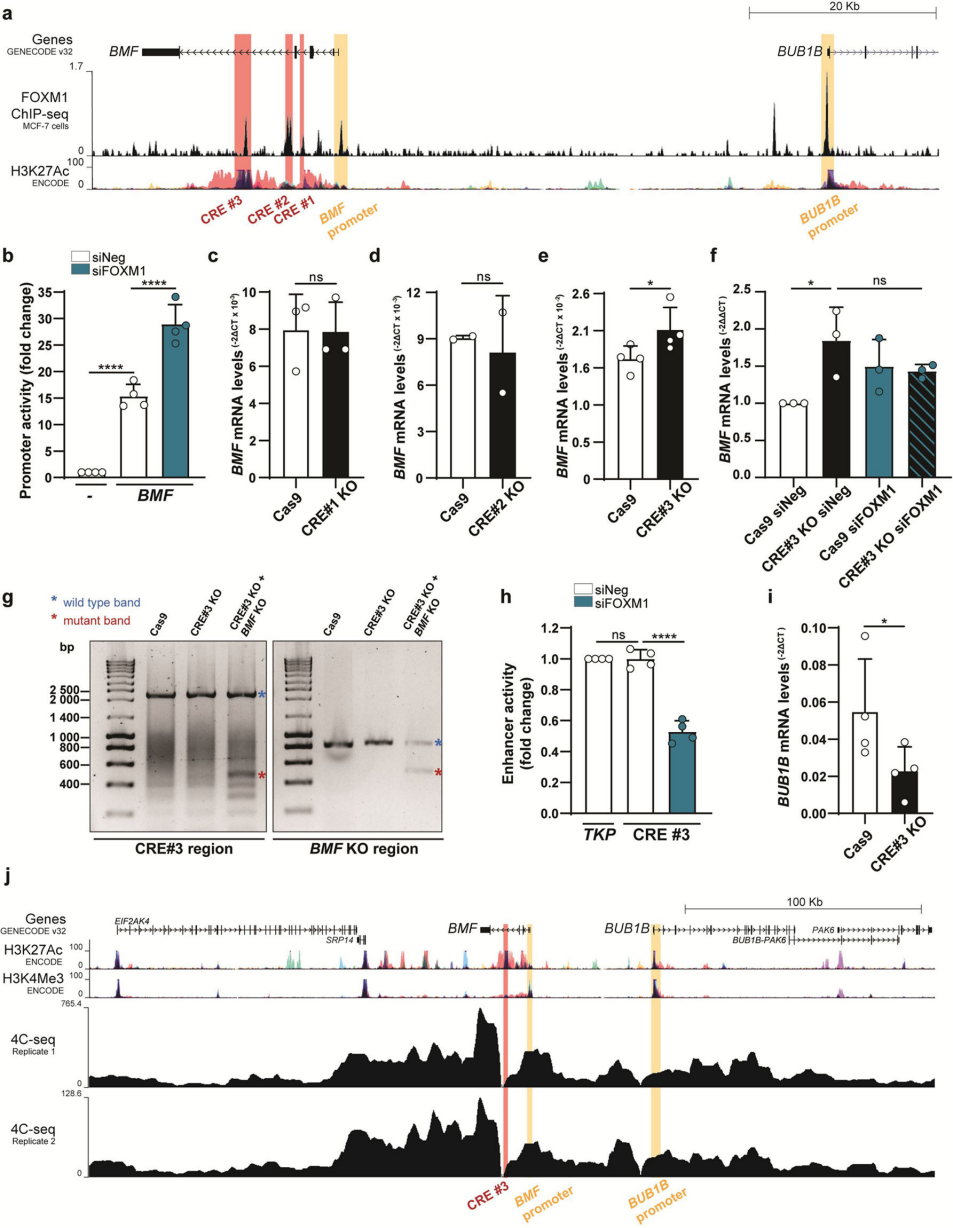
FOXM1-binding CRE within *BMF* intronic region represses *BMF* while enhancing *BUB1B* expression. **a** *BMF* and *BUB1B* genomic landscape from UCSC genome browser with tracks of FOXM1^32^ and H3K27Ac^33^ ChIP-seq data. **b** *BMF* promoter-reporter assay in siNeg- and siFOXM1-depleted MCF-7 cells. **c** *BMF* transcript levels in CRE#1 CRISPR/Cas9-deleted MCF-7 polyclonal cell population. **d** *BMF* transcript levels in CRE#2 CRISPR/Cas9-deleted MCF-7 polyclonal cell population. **e** *BMF* transcript levels in CRE#3 CRISPR/Cas9-deleted MCF-7 cells immediately after sorting. **f** *BMF* transcript levels in CRE#3 CRISPR/Cas9-deleted and siFOXM1-depleted MCF-7 cells immediately after sorting. **g** Genomic PCR validation for the ability to generate CRE#3 CRISPR/Cas9-deleted polyclonal cell population only in a *BMF* KO background. **h** Enhancer reporter assay for CRE#3 in siNeg- and siFOXM1-depleted MCF-7 cells. **i** *BUB1B* transcript levels in CRE#3 CRISPR/Cas9-deleted MCF-7 cells immediately after sorting. **j** 4C-seq replicates of neonatal HDFs with a viewpoint in CRE#3 (red). *BMF* promoter and *BUB1B* promoter interactions with CRE#3 are highlighted (orange). Data information: in **b**, **f**, **h** data are mean ± S.D. from *n* = 3 or *n* = 4 independent experiments; ****p ≤ 0.0001 (two-tailed one-way ANOVA and Tukey’s multiple comparison test). In **c**, **d** data are mean ± S.D. from *n* = 3 and *n* = 2 independent experiments; ns, *p* ≤ 0.05 (two-tailed Mann–Whitney test). In **e**, **i** data are mean ± S.D. from *n* = 4 independent experiments; *p ≤ 0.05 (two-tailed paired *t* test).

To gain insight into the mechanism by which FOXM1 represses *BMF* transcription during mitosis, we searched for potential FOXM1-binding sites in the *BMF* genomic region. Interrogation of a publicly available FOXM1 ChIP-seq data set for MCF-7 cells^32^ revealed the *BMF* promoter, as well as several intronic regions with an H3K27ac mark for active CREs^33^, as FOXM1-binding sites (Fig. 4a). To assess the role of FOXM1 binding at the *BMF* promoter, we performed a promoter-luciferase reporter assay in MCF-7 siNeg- and siFOXM1-depleted cells. We found *BMF* promoter activity in comparison with negative control in siNeg-depleted cells, further enhanced upon siRNA-mediated depletion of FOXM1 (Fig. 4b). This FOXM1 transcriptional repression at the *BMF* promoter is not through promoter methylation as previously reported for *GATA3*, in which FOXM1 was shown to recruit the DNA methylase DNMT3B^34^ (Fig. S6a). Also, we excluded transcriptional activation by FOXO3 as a competing mechanism. Although *BMF* has been described as FOXO3 transcriptional target^21^, and FOXO3 and FOXM1 to function antagonistically in cancer cells^35^, we found FOXO3 depletion to induce *BMF* upregulation and *FOXM1* downregulation in HDFs (Fig. S6b).

As CREs can modulate gene expression, we deleted the putative CREs#1–3 (Fig. 4a) using CRISPR/Cas9-mediated genomic editing. CRE#1 and CRE#2 deletions did not change *BMF* gene expression in polyclonal MCF-7 cell populations (Fig. 4c, d; Fig. S6c, d). Regarding CRE#3 deletion, we repeatedly failed to establish polyclonal cultures from CRE#3-deleted cells (Fig. S6e). This was not due to single guide RNAs (sgRNAs) inefficacy, as successful genomic deletion was detected immediately after fluorescence-activated cell sorting (FACS) (Fig. S6f). Importantly, qPCR analysis in these FACS-sorted CRE#3-deleted cells revealed *BMF* upregulation (Fig. 4e), thus establishing this FOXM1-binding CRE as a transcriptional *BMF* silencer. In addition, we confirmed that FOXM1 RNAi, CRE#3 genomic deletion, and their combination, all exhibit equivalent effect in *BMF* upregulation, which supports that FOXM1 represses *BMF* through its binding to CRE#3 (Fig. 4f). To confirm that cell death driven by *BMF* upregulation, and not *BUB1B* downregulation, was precluding the establishment of a polyclonal cell population deleted for CRE#3, we performed the CRISPR/Cas9-mediated CRE#3 deletion in *BMF KO* (Fig. 4g) and in *BUB1B OX* (Fig. S6g–i) MCF-7 cells. Under the *BMF KO* genetic background, but not under *BUB1B OX*, we were able to establish a polyclonal cell population deleted for CRE#3 (Fig. 4g), which demonstrates that CRE#3 is required for cell viability. Overall, our data disclosed the FOXM1-binding CRE#3 as a transcriptional *BMF* silencer.

### CRE within *BMF* intronic region works bifunctionally as *BUB1B* enhancer and *BMF* silencer to inhibit anoikis during prolonged mitosis

To gain further insight into the functional role of CRE#3, and considering the presence of the H3K27ac mark from the available ChIP-seq data set^33^, we performed an enhancer-luciferase reporter assay. We found equivalent enhancer activity in cells transfected with the CRE#3-containing reporter plasmid in comparison with the positive control (Tyrosine kinase promoter) (Fig. 4h). Moreover, FOXM1 repression reduced the CRE#3 regulatory activity. Interestingly, we noticed that in the vicinity of the *BMF* genomic region is the *BUB1B* spindle assembly checkpoint gene, an established FOXM1 transcriptional mitotic target. qPCR analysis of CRISPR/Cas9 CRE#3-deleted cells immediately after FACS revealed decreased *BUB1B* transcript levels (Fig. 4i), supporting that CRE#3 is a *BUB1B* enhancer. It is known that co-regulated genes are normally confined in close proximity and that CREs and promoters are brought together by chromatin looping^36^. To ascertain chromatin interactions between CRE#3 and the *BMF* and *BUBR1* promoter regions, we performed 4C-sequencing analysis with the viewpoint on CRE#3. We found that *BMF* promoter, *BUB1B* promoter, and CRE#3 physically interact (Fig. 4j). Thus, our data disclose an unforeseen FOXM1-binding CRE that acts to induce *BUB1B* transcription while repressing *BMF* expression. We propose that through this mechanism, mitotic cells are able to circumvent cell death signaling induced by prolonged mitotic arrest.

### High *BMF* levels correlate with response to anoikis-inducing chemotherapeutic drugs

**Fig. 5:**
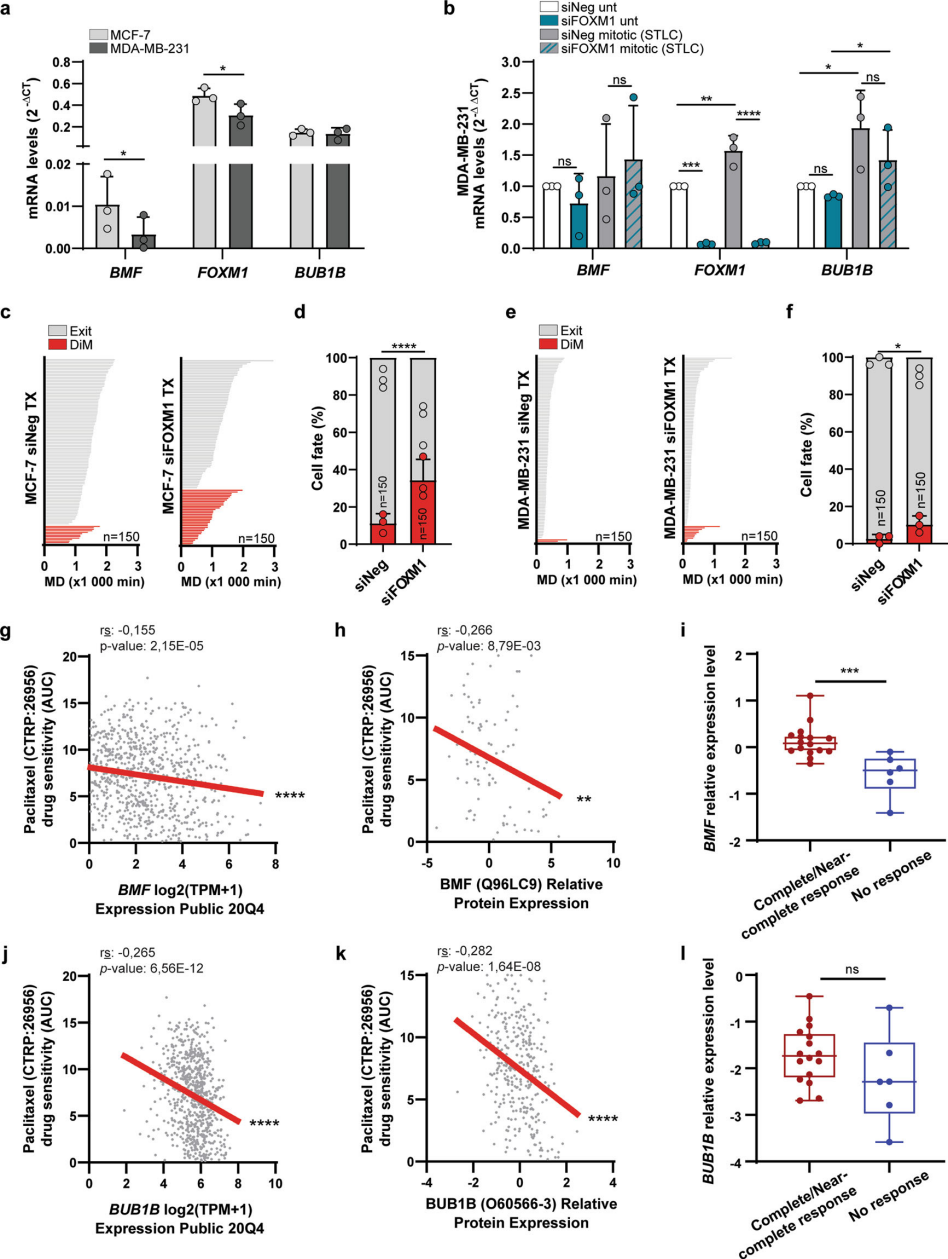
*BMF* expression correlates with response to antimitotic drug chemotherapy. **a** *BMF*, *FOXM1,* and *BUB1B* transcript levels in MCF-7 and MDA-MB-231 cells. **b** *BMF*, *FOXM1,* and *BUB1B* transcript levels in asynchronous (untreated, unt) and mitotic (shake-off upon STLC) MDA-MB-231 cells depleted with Neg or FOXM1 siRNAs. **c** Individual cell fate profiling of siNeg- (*n* = 150) or siFOXM1-depleted (*n* = 150) MCF-7 cells treated with 500 nM TX. *MD* mitotic duration. **d** Cell fate percentage of the experiments described in **c**. **e** Individual cell fate profiling of siNeg- (*n* = 150) or siFOXM1-depleted (*n* = 150) MDA-MB-231 cells treated with 500 nM TX. MD, mitotic duration. **f** Cell fate percentage of the experiments described in **e**. **g** Correlation of *BMF* mRNA levels and paclitaxel drug sensitivity in the CTRP drug response data set. **h** Correlation of BMF protein levels and paclitaxel drug sensitivity in the CTRP drug response data set. **i** Relative *BMF* mRNA expression levels in tumors with complete/near-complete response vs. no response to a combination of docetaxel and capecitabine^40^. **j** Correlation of *BUB1B* mRNA levels and paclitaxel drug sensitivity in the CTRP drug response data set. **k** Correlation of BUB1B protein levels and paclitaxel drug sensitivity in the CTRP drug response data set. **l** Relative *BUB1B* mRNA expression levels in tumors with complete/near-complete response vs. no response to a combination of docetaxel and capecitabine^40^. Data information: In **a** data are mean ± S.D. from *n* = 3 independent experiments; *p ≤ 0.05 (two-tailed paired *t* test). In **b** data are mean ± S.D. from *n* = 3 independent experiments; *p ≤ 0.05, **p ≤ 0.01, ***p ≤ 0.001, ****p ≤ 0.0001 (two-tailed one-way ANOVA and Tukey’s multiple comparison test). In **d**, **f** data are mean ± S.D. of *n* = 3 independent experiments; *p ≤ 0.05, ****p ≤ 0.0001 (two-tailed *χ*^2^). In **g**, **h**, **j**, **k** **p ≤ 0.01, **** *p* ≤ 0.0001 (https://depmap.org/portal/interactive/). In **i**, **l** ***p ≤ 0.001, ns *p* > 0.05 (two-tailed Mann–Whitney test).

To extend our findings into antimitotic chemotherapy response, we investigated the FOXM1-dependent co-regulation of *BMF* and *BUB1B* expression in the context of tumor cell treatment. As a preliminary approach, we used the MCF-7 and MDA-MB-231 breast cancer cell lines reported as anoikis-sensitive and anoikis-resistant, respectively^21^. In agreement, we confirmed higher expression of *BMF* in MCF-7 vs. MDA-MB-231 cells, even though *FOXM1* and *BUB1B* transcript levels were similar in both cell lines (Fig. 5a). We then evaluated if *BMF* expression is cell cycle-regulated in a FOXM1-dependent manner in MDA-MB-231 cells, as we did for the MCF-7 cells (see Fig. 3c). Interestingly, qPCR analysis of mitotic vs. asynchronous MDA-MB-231 cell populations revealed no significant changes in *BMF* expression, even upon FOXM1 RNAi (Fig. 5b). Accordingly, time-lapse imaging of siNeg- and siFOXM1-depleted MCF-7 and MDA-MB-231 cells treated with paclitaxel confirmed a higher resistance of MDA-MB-231 cells in comparison with MCF-7 cells as described^37^, and revealed that FOXM1 repression only modestly increases DiM in the MDA-MB-231 anoikis-resistant cells in comparison with the MCF-7 cells (Fig. 5c–f). Similar results were obtained when using small-molecule inhibition of FOXM1 with FDI-6 (Fig. S7a–d). Conversely, we also confirmed FOXM1 overexpression in MDA-MB-231 cells not to change BMF expression (Fig. S7e, f). Thus, these data suggest that anoikis-resistant cells are able to repress *BMF* in a FOXM1-independent manner. Still, CRISPR/Cas9-mediated CRE#3 deletion impairs MDA-MB-231 cell viability and induces *BMF* upregulation (Fig. S7g–i), suggesting that CRE#3 is required for *BMF* repression, although not through FOXM1 as in MCF-7 cells.

To examine *BMF* expression in a wider tumor context, we interrogated the Cancer Cell Line Encyclopedia (CCLE)^38^ for the association between mRNA and protein expression levels with drug responses in the Cancer Target Discovery and Development (CTD^2^) database^39^, specifically for chemotherapeutics most extensively reported as anoikis-inducing: paclitaxel, docetaxel, and doxorubicin. We found a significant correlation of higher *BMF* mRNA and protein levels with response to these drugs (Fig. 5g, h; Table S1). Interestingly, higher *BUB1B* mRNA and protein levels also correlated with increased drug sensitivity as observed for *BMF*, suggesting the disruption of the FOXM1-dependent co-regulatory mechanism found in untransformed and MCF-7 cells (Fig. 5j, k; Table S1). Accordingly, *FOXM1* mRNA and protein levels did not show a correlation with any drug (Table S1). Thus, this analysis supports that tumor cells with lower *BMF* expression are more resistant to anoikis-inducing drugs, and independently of FOXM1 expression.

To further examine the clinical relevance of these findings, we interrogated microarray data sets from XeNA, a clinical trial examining response rates in women with operable, early-stage breast cancer receiving neoadjuvant capecitabine plus the antimitotic agent docetaxel^40^. Tumors from patients showing no response had diminished *BMF* expression (Fig. 5i). Consistent with the premise that *BMF* expression in these non-responsive patients is FOXM1-independent, we found no significant elevated expression in the case of *BUB1B* (Fig. 5l).

## Discussion

**Fig. 6:**
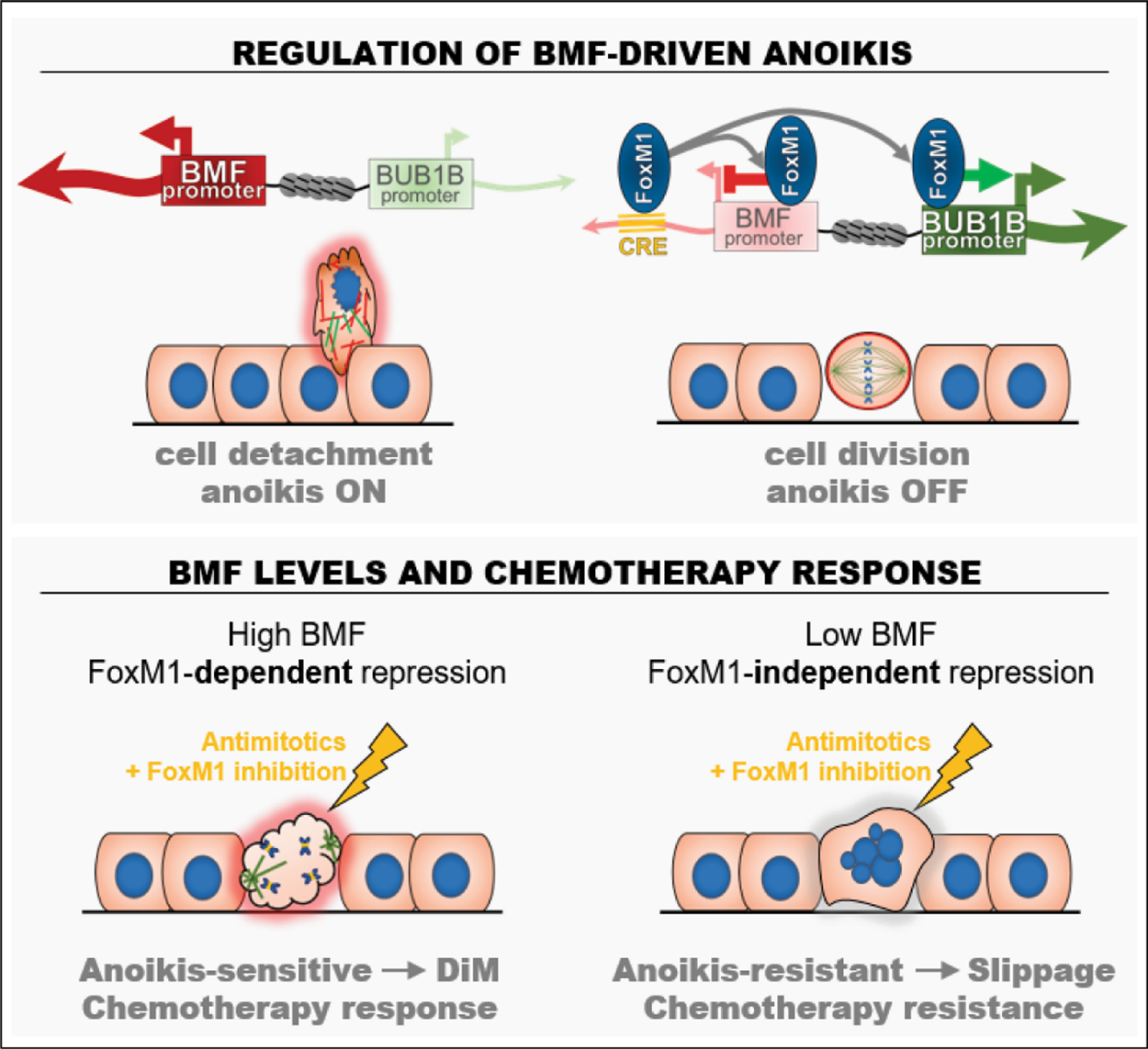
Working model integrating *BMF* transcriptional regulation by FOXM1 in mitosis and antimitotic chemotherapy. Upper panel: FOXM1 inhibits *BMF* expression and anoikis induction during prolonged mitosis. A FOXM1-binding *cis*-regulatory element (CRE) in *BMF* is an enhancer of the *BUB1B* neighbor mitotic gene. Lower panel: FOXM1 inhibition increases DiM in response to antimitotic drugs in anoikis-sensitive cells (high BMF). FOXM1-independent *BMF* repression (low BMF) correlates with resistance to antimitotic chemotherapy.

Our study brings insight into the poorly understood mechanisms that define the kinetics of death signaling accumulation and threshold during mitotic arrest induced by antimitotic chemotherapy. We show that FOXM1 transcriptional activity driving G2/M cell cycle gene expression, also modulates the apoptotic pathway of the competing networks model. Both genetic and pharmacological repressions of FOXM1 increased DiM upon antimitotic drug treatment. Previous studies have reported a correlation between FOXM1 repression and paclitaxel response, with targets including genes involved in MT dynamics or drug efflux^8–12^. Here, we originally identified an apoptotic gene repressed by G2/M FOXM1 transcriptional activity, *BMF*. FOXM1 binds to the *BMF* promoter and an intronic CRE#3 to inhibit its expression. Interestingly, we found that transcription of the *BMF* neighbor gene, *BUB1B*, which is a FOXM1 transcriptional target, is coupled with *BMF* repression. We showed that the *BMF* promoter, *BUB1B* promoter, and CRE#3 physically interact, with the FOXM1-binding CRE operating bifunctionally to induce *BUB1B* transcription, whereas silencing *BMF* (Fig. 6). Curiously, this resembles an antithetical regulation of endothelial *ACE* and *ACE2* by a BRG1-FOXM1 complex previously reported in the context of pathologic cardiac hypertrophy^41^.

Importantly, the transcriptional regulation here described explains how cells with low FOXM1 levels become more prone to DiM upon antimitotic drug treatment. *BMF* genomic deletion was able to revert increased DiM under low FOXM1 transcriptional activity, disclosing anoikis as an unforeseen apoptotic program in response to antimitotics. This supports the idea of using FOXM1 inhibition to improve antimitotic drug response. However, when extrapolating our findings into a wide tumor context, we found that FOXM1-dependent *BMF* repression appears disrupted in anoikis-resistant cells. Interrogation of the CCLE for the association between *FOXM1* and *BMF* expression levels with anoikis-inducing drug responses in the CTD^2^ database revealed a significant correlation only for *BMF*. In agreement, we found FOXM1-independent *BMF* repression in the anoikis-resistant MDA-MB-231 cell line, whereas FOXM1 inhibition still upregulated *BMF* and increased DiM in the anoikis-sensitive MCF-7 cell line. Although the detailed mechanism of FOXM1-independent *BMF* repression remains unclear, we found that CRE#3 is still essential. Importantly, our findings disclose *BMF* levels as predictive of tumor response to antimitotic chemotherapy, with only higher levels supporting combination therapy of FOXM1 inhibitors with antimitotics (Fig. 6).

Other mechanisms have been reported to influence the timing of mitotic cell death, such as degradation of Mcl-1^42^, Cdk1-dependent caspase phosphorylation^43^, c-Myc overexpression^44^, and cGAS activation^45^. Whether these mechanisms cross-talk with the FOXM1 transcriptional competence and BMF apoptotic potential remains unknown. FOXM1 has been involved in regulatory loops with Cdk1^46,47^ and c-Myc^48,49^, with Cdk1 and c-Myc upregulating FOXM1 activity, as well as the other way round. One interesting aspect about FOXM1 is that it regulates targets of the two competing networks determining fate decision upon mitotic arrest, with this study disclosing its role in the apoptotic pathway. Further investigation on how FOXM1 transcriptional activity becomes uncoupled from BMF-driven apoptosis will contribute to our understanding of chemotherapy response.

## Methods and materials

### Cell culture

HDFs from healthy Caucasian males were obtained from the Coriell Cell Repository (NJ, USA) (HDF 10 y GM03348, HDF 87 y AG10884) and Zen Bio (NC, USA) (HDF N DFM021711A) and kept at a low passage number. HDFs were cultured in minimal essential medium Eagle–Earle (MEM) supplemented with 15% fetal bovine serum (FBS), 2.5 mM l-glutamine, and 1× antibiotic–antimycotic (all from Gibco, Thermo Fisher Scientific, CA, USA). MCF-7 cells (ATCC HTB-22) and MDA-MB-231 (HTB-26) were obtained from ATCC (VA, USA) and cultured with Dulbecco’s modified Eagle’s medium GlutaMAX (DMEM) (Gibco, Thermo Fisher Scientific, CA, USA) supplemented with 10% FBS and 1× antibiotic–antimycotic. All cells were grown in ventilated flasks at 37 °C and humidified atmosphere with 5% CO_2_.

### Drug treatments

*S*-Trityl-l-cysteine (STLC, Tocris, UK) was used at 5 µM, and paclitaxel (TX, Sigma-Aldrich, MO, USA) at 500 nM. For mitotic shake-off, cells were treated with antimitotic drugs for 16 h. For live-cell imaging, cells were treated for 24 h with FDI-6 (Axon Medchem, VA, USA) and RCM-1 (kindly provided by Vladimir Kalinichenko, OH, USA). In all, 1 mM thymidine block for 20 h followed by a 7 h washout into the fresh medium was used for G2 cell synchronization.

### RNA interference

HDFs were transfected with 45 nM siRNA Universal Negative Control #1 (Sigma-Aldrich, MO, USA), 45 nM FOXM1 small interfering RNA (SASI_Hs01_00243977, Sigma-Aldrich, MO, USA), or 45 nM FOXO3 small interfering RNA (SASI_HS01_00119127, Sigma-Aldrich, MO, USA) using Lipofectamine RNAiMAX (Thermo Fisher Scientific, CA, USA) according to manufacturer’s instructions. MCF-7 and MDA-MB-231 were transfected with 20 nM siRNA. Cells were analyzed 72 h post transfection, and depletion efficiency was validated by qPCR.

### Plasmid transfection

HDFs were transfected with 500 ng of pcDNA3/Cyclin B1-GFP (#39871, Addgene) using Lipofectamine 3000 (Life Technologies, Thermo Scientific, CA, USA) according to the manufacturer’s instructions. Cells were analyzed 48 h post transfection.

### Lentiviral production and infection

pLVX-FOXM1-dNdK was generated as previously described in ref. ^27^. pBabe HA human BMF plasmid (#17239, Addgene) was digested with *BamH*I and *EcoR*I and the coding sequence inserted into the pLVX-Tight-Puro Vector (PT3996-5, Clontech, Takara Bio USA) to generate the pLVX-BMF-Flag construct. *BUB1B* coding sequence was reverse-transcribed from HDF N total RNA and inserted into pLVX-Tight-Puro. Lentiviruses were produced in HEK293T cells transfected with pLVX-empty, pLVX-BMF-Flag, pLVX-BUB1B, pLVX-FOXM1-dNdK or pLVX–Tet-On Advanced (PT3990-5, Clontech, Takara Bio USA) and the packaging plasmids (pMd2.G and psPAX2, #12259 and #12260, respectively, Addgene) using Lipofectamine 2000 (Life Technologies, Thermo Scientific, CA, USA) according to manufacturers’ instructions. Cells were transduced with inducible and transactivator viruses at 2:1 ratio in media containing 8 µg/ml polybrene (Sigma-Aldrich, MO, USA). 500 ng/ml doxycycline (Sigma-Aldrich, MO, USA) was added to fresh media and phenotypes analyzed 24 h later for BMF OX and 72 h later for FOXM1 OX. Transduction efficiency was measured by Flag immunostaining or by *FOXM1/BUB1B* qPCR. 1 µg/mL puromycin and 400 µg/mL G418 antibiotic selection was used to enrich MCF-7 BUB1B OX, MCF-7 BMF OX and MDA-MB-231 FOXM1 OX cells.

### CRISPR/Cas9-mediated deletions and validation

sgRNAs upstream and downstream of the target region were designed using the Benchling online platform (www.benchling.com) (see Table S2), and inserted in pSpCas9(BB)-2A-GFP (PX458) (#48138, Addgene) and pU6-(*Bbs*I) CBh-Cas9-T2A-mCherry (#64324, Addgene) plasmids digested with *Bbs*I. Cells were plated in six-well plate and transfected with 500 ng of each plasmid using Lipofectamine 3000 (Life Technologies, Thermo Scientific, CA, USA) according to manufacturers’ instruction. After 72 h, cells were sorted in a FACSAria II Cell Sorter (BD Biosciences, CA, USA) using an 85 μm nozzle and the laser lines 488 nm and 561 nm. Cells were gated by forward scatter area (FSC-A) vs. side scatter area and FSC-A vs. FSC-width plot to exclude dead cells and doublets/clumps, respectively. The positive gates were established based on autofluorescence control. GFP/mCherry-positive cells were either collected into a 24-well plate to establish polyclonal cell cultures or collected directly for qPCR analysis. DNA deletions were validated immediately after FACS (1000 cells) or upon the establishment of polyclonal cell cultures. DNA was extracted by boiling the samples in Tris-HCl pH 8.0 for 15 min, incubating with 1 µg/µl proteinase K at 56 °C for 30 min, and inactivating at 95 °C for 5 min. Genotyping was performed with i-MAX II DNA Polymerase (High Fidelity) (iNtRON Biotechnology, South Korea) according to manufacturers’ instructions. The primers used are listed in Table S3.

### Time-lapse live-cell imaging

Cells were plated in four-well or eight-well µ-slides (Ibidi, Germany). Time-lapse images were either acquired in a Leica DMI6000b (Leica Microsystems, Germany) or in a Zeiss Axiovert 200 M (Carl Zeiss, Oberkochen, Germany) inverted microscope. All experiments were done under controlled temperature, atmosphere, and humidity. Neighbor fields were imaged every 5–10 min for 48–72 h. Images were then analyzed in ImageJ/Fiji software.

### Western blotting

Western blotting was performed as previously described in ref. ^27^.

### Apoptosis assay

Apoptosis was assessed by flow cytometry using the BD Accuri C6 (BD Biosciences, CA, USA) and the (FITC)-conjugated Annexin V/Apoptosis detection kit (BioLegend, Inc. San Diego, CA, USA). Data were analyzed using the FlowJo v10 software and the gates defined accordingly to the autofluorescence control.

### Immunostaining

Cells were fixed in 4% paraformaldehyde in PBS for 20 min, permeabilized with 0.3% Triton-X100 in PBS for 7 min, blocked in 10% FBS, and incubated overnight at 4 °C with mouse anti-Flag M2 (Sigma-Aldrich, MO, USA) diluted in 5%FBS/PBS. Cells were then incubated for 1 h at room temperature with the secondary antibody AlexaFluor-568 (Life Technologies, CA, USA), and DNA was counterstained with 1 µg/ml DAPI (Sigma-Aldrich, MO, USA). Images were acquired in a Zeiss AxioImager Z1 (Carl Zeiss, Oberkochen, Germany) and analyzed using ImageJ/Fiji software.

### Quantitative real-time PCR

Total RNA was extracted using Quick-RNA Microprep Kit (Zymo Research, CA, USA). cDNA synthesis was performed using NZY First-Strand cDNA Synthesis Kit (NZYtech, Portugal). qPCR analysis was performed in a CFX384 Touch Real-Time PCR Detection System using iTaq Universal SYBR Green Supermix accordingly to manufacturers’ instruction, and data analyzed using the CFX Maestro Software (all from Bio-Rad Laboratories, CA, USA). The 2^−∆Ct^ or 2^−∆∆Ct^ methods were used to quantify the transcript levels against three housekeeping genes (*GAPDH, HPRT1*, T*BP*). The primers used are listed in Table S4.

### Cell cycle profiling

Cells were harvested, centrifuged at 1500 rpm for 5 min, and rinsed in ice-cold PBS. After centrifugation, cells were resuspended in 500 µL of ice-cold PBS. An equal volume of 75% cold ethanol was added drop-by-drop, while gently vortexing. Samples were chilled on ice for 30 min. Fixed cells were centrifuged at 2000 rpm for 5 min, resuspended in PBS, and incubated with RNase A for 3 h at 37 °C. Propidium iodide was used at 50 µL/mL for DNA counterstaining. Cells were filtered and the cell cycle profile was assessed by flow cytometry using the BD Accuri C6 (BD Biosciences, CA, USA). Data were analyzed using the FlowJo v10 software.

### Bisulphite sequencing

The EpiTect Bisulphite Kit (Qiagen, DE) was used accordingly to the manufacturers’ instructions. The primers used are listed in Table S5.

### Luciferase activity assays

The *BMF* promoter and CRE#3 regions were amplified from HDF N genomic DNA by PCR. The *BMF* promoter amplicon was cloned into pNL1.1 plasmid [Nluc] (Promega #N1441, WI, USA) to generate Nluc/BMF. The CRE#3 amplicon was inserted into pCR8/GW/TOPO vector and then cloned into pGL4.23-GW plasmid (Addgene #60323) via Gateway cloning. siNeg- and siFOXM1-depleted MCF-7 cells were used for the luciferase reporter assays. For the promoter-reporter assay, cells were transfected with 3 μg of pGL4.54 [luc2/TK] (Promega #E5061, WI, USA) and 300 ng of either pNL1.1 (negative control) or NLuc/BMF using Lipofectamine 3000 (Life Technologies, Thermo Scientific, CA, USA) accordingly to manufacturers’ instruction. For the enhancer reporter assay, cells were transfected with 30 ng of Nluc and 3 μg of either pGL4.23-GW (negative control), luc2/TKp (positive control), or luc2/CRE#3 plasmid. pGL4.54 [luc2/TK] and pNL1.1 [Nluc] plasmids were used as transfection controls in the promoter and enhancer assays, respectively. Data were acquired using the Nano-Glo® Dual-Luciferase® Reporter Assay System kit (Promega, WI, USA) in a Synergy™ 2 Multi-Mode Microplate Reader (BioTek, VT, USA). The luciferase activity was normalized to the activity of the transfection control and is presented as a relative response ratio, in which the responses to the empty vector and positive control vector were normalized to 0 and 1, respectively.

### 4C-sequencing

10^6^ HDF N cells were used for 4C-seq analysis as previously described^50^ with minor adaptations. Restriction digestions were done with the enzymes *DpnII* and *Csp6I*. The final template was purified using an Amicon Ultra-15 Centrifugal Filter Device (Millipore, MA, USA). Two libraries were independently prepared with the Expand Long Template polymerase (Roche, CH) using primers with adaptors (Table S6). The resulting 4C4C libraries were purified with the QIAquick PCR Purification kit (QIAGEN, DE) followed by the Agencourt AMPure XP reagent (Beckman Coulter, CA, USA). Libraries were sequenced on an Ion S5XL System (Ion Torrent, Thermo Fisher Scientific, MA, USA). Processing and alignment of the sequencing data to the human genome (hg38) were performed as previously described^51^.

### Association of drug sensitivities with gene expression levels

The mRNA and protein expression levels were obtained from CCLE mRNA expression (11/2020 DepMap release; CCLE_Expressionl.Full.csv) and CCLE proteomics (01/2020 release; protein_quant_current_normalized.csv) data sets, respectively. The drug sensitivity levels were obtained from the CTRP CTD^2^ data set (12/2015 DepMap release; CTRPv2.0_2015_ctd2_ExpandedDataset.zip). Spearman’s correlation coefficient values and linear regression p-values were calculated using the default association analysis incorporated in the DepMap portal (https://depmap.org/portal/interactive/). The mRNA expression levels of clinical samples were obtained from the Gene Expression Omnibus portal (accession number GSE22358)^40^. The relative expression levels of *BMF* (probe ID: 37059) and *BUB1B* (probe ID: 20572) were compared between the tumors with the complete or near-complete response and those with no response to a combination of docetaxel and capecitabine, as was previously done for other genes^44^. The statistical analysis between the groups was performed using a two-tailed Mann–Whitney test, in GraphPad Prism 9.0 (GraphPad, CA, USA).

### Statistical analysis

Statistical analysis was performed using GraphPad Prism 8.0 (except association of drug sensitivities with gene expression levels analysis). Sample sizes and statistical tests are indicated in the figure legends for each experiment. Parametric and non-parametric tests were applied adequately. For pairwise comparisons, either a two-tailed *t* test, two-tailed Mann–Whitney, or two-tailed *χ*^2^-square was used, otherwise, two-tailed one-way analysis of variance was applied followed by a post hoc for multiple comparisons test. ns, *p* > 0.05, *p ≤ 0.05, **p ≤ 0.01, ***p ≤ 0.001, and ****p ≤ 0.0001. Values are shown as mean ± S.D.

## Supplementary information

Supplemental material Figure and Table Legends

Figure S1

Figure S2

Figure S3

Figure S4

Figure S5

Figure S6

Figure S7

Table S1

Table S2

Table S3

Table S4

Table S5

Table S6

## Data Availability

The 4C-seq data sets for this publication were deposited in the European Nucleotide Archive (ENA) at EMBL-EBI under the accession number PRJEB44770 (https://www.ebi.ac.uk/ena/browser/view/PRJEB44770).
