# Supplementary material for: FOXM1 repression increases mitotic death upon antimitotic chemotherapy through BMF upregulation
Source: Cell Death Dis. 2021 May 25;12(6):542. doi: 10.1038/s41419-021-03822-5 (PMC8149823; doi:10.1038/s41419-021-03822-5)
Supplement: Supplementary file 1 — Supplemental material Figure and Table Legends [file 41419_2021_3822_MOESM1_ESM.docx]

**SUPPLEMENTAL MATERIAL FIGURE LEGENDS**

**Figure S1. Modulation of FOXM1 expression levels in HDFs impact cell fate profiling in response to antimitotics. a)** Western blot analysis of FOXM1 protein levels in 87y HDFs transduced with empty and pLVX-FOXM1-dNdK plasmids. **b)** Western blot analysis of FOXM1 protein levels in mock and siFOXM1-depleted 10y HDFs. **c)** Individual cell fate profiling (exit vs. DiM) of control (empty vector) (n=180) and FOXM1-overexpressing (n=116) 87y HDFs treated with 500nM paclitaxel (TX). MD, mitotic duration. **d)** Cell fate percentage of the experiments described in c. **e)** Mitotic duration of the individual cell fates shown in c. **f)** Individual cell fate profiling of mock- (n=130) and siFOXM1-depleted (n=82) 10y HDFs treated with 500nM TX. MD, mitotic duration. **g)** Cell fate percentage of the independent experiments described in f. **h)** Mitotic duration of the individual cell fates shown in f. **i)** Cell cycle profile of siNeg- and siFOXM1-depleted young HDFs, treated with DMSO, STLC or TX. **j)** Individual cell fate profiling of control (empty) or FOXM1 overexpressing (FOXM1 OX) neonatal HDFs, depleted with Neg or FOXM1 siRNAs, and treated with 5µM STLC. MD, mitotic duration. n=cell number. **k)** Cell fate percentage of the experiments described in j.

Data information: In a, b data are representative images of n=3 independent experiments. In d, g, k data are mean ± S.D. of n=2 independent experiments; ns *p*>0.05, ** *p*≤0.01, **** *p*≤0.0001 (two-tailed χ2). In e, h mean values are indicated; ns *p*>0.05, * *p*≤0.05, ** *p*≤0.01 (two-tailed Mann-Whitney test). In i data are mean ± S.D. from n=3 independent experiments; ns *p*>0.05 (two-tailed two-way ANOVA and Tukey multiple comparison test).

**Figure S2. Pharmacological modulations of FOXM1 activity impact cell fate profiling in response to paclitaxel. a)** Representative phase-contrast and fluorescent microscopy time-lapse sequences of a neonatal fibroblast expressing cyclin B1-GFP and treated with 5µm STLC. Time in hr:min, starting at NEB. Scale bar, 20µm. **b)** Cyclin B1–GFP levels decay in young (HDF N, n=7) and elderly (HDF 87y, n=6) fibroblasts during prolonged mitotic arrest. For each cell, timepoint fluorescence intensity levels were normalized to the levels at NEB (time 0). **c)** Individual cell fate profiling of neonatal HDFs treated with 500nM TX in the absence (DMSO, n=161) or presence of FOXM1 inhibitor (RMC-1, n=136). MD, mitotic duration. **d)** Cell fate percentage of the experiments described in i. **e)** Mitotic duration of the individual cell fates shown in i. **f)** Individual cell fate profiling of neonatal HDFs treated with 500nM TX in the absence (DMSO, n=250) or presence of FOXM1 inhibitor (FDI-6, n=250). MD, mitotic duration. n=cell number. **g)** Cell fate percentage of the experiments described in l. **h)** Mitotic duration of the individual cell fates shown in l.

Data information: In b values are arbitrary units (A.U.) mean ± S.D.. In d, g data are mean ± S.D. of n=3 independent experiments; **** *p*≤0.0001 (two-tailed χ2). In e, h mean values are indicated; ns *p*>0.05, * *p*≤0.05, ** *p*≤0.01 (two-tailed Mann-Whitney test).

**Figure S3. BMF expression accounts for increased DiM in response to paclitaxel. a)** Representative image of neonatal HDFs expressing Flag-tagged BMF (in red). Scale bar, 20µm. **b)** Percentage of cells expressing Flag-tagged BMF upon plasmid transfection. **c)** Individual cell fate profiling of control (empty vector) (n=300) and BMF-expressing neonatal HDFs (n=300) treated with 500nM TX. MD, mitotic duration. **d)** Cell fate percentage of the experiments described in c. **e)** Mitotic duration of the individual cell fates shown in c. **f)** CRISPR/Cas9 gene edition layout for the deletion of exon III in human *BMF.* Upstream and downstream sgRNA plasmids expressing GFP and mCherry, respectively, were used to allow cell sorting of double positive cells (in yellow). Sorted cells were used for establishment of a polyclonal cell culture. **g)** Validation by PCR of the efficiency of *BMF* CRISPR/Cas9 genomic deletion in the polyclonal cell population (*BMF* KO*).* **h)** Individual cell fate profiling of siNeg- or siFOXM1-depleted *BMF* KO HDFs treated with 500nM TX. MD, mitotic duration. **i)** Cell fate percentage of the experiments described in h. **j)** Mitotic duration of the individual cell fates shown in h.

Data information: In b values are mean ± S.D. of n=4 independent experiments. In d, i values are mean ± S.D. of n=3 independent experiments; *** *p*≤0.001, **** *p*≤0.0001 (two-tailed χ2). In e mean values are indicated; **** *p*≤0.0001 (two-tailed Mann-Whitney test). In j mean values are indicated; * *p*≤0.05, ** *p*≤0.01, *** *p*≤0.001, **** *p*≤0.0001 (two-tailed Kruskal-Wallis and Dunn’s multiple comparison test).

**Figure S4. FOXM1 depletion or BMF overexpression do not instigate mitotic death during timely mitosis in the absence of antimitotic drugs. a)** Representative time-lapse sequences of HDF bipolar divisions (blue) under the different experimental conditions. Time in hr:min, from nuclear envelope breakdown to anaphase onset. Scale bar, 20µm. **b)** Individual cell fate profiling of mock (n=30) or siFOXM1-depleted (n=30) 10y HDFs. MD, mitotic duration. Exit/slippage and DiM were never observed.

**c)** Individual cell fate profiling of HDFs transduced with empty pLVX (n=40) or pLVX-BMF (n=40) plasmids. MD, mitotic duration. Exit/slippage and DiM were never observed.

**Figure S5. Cell cycle regulation of *BMF* expression. a)** Cell cycle cytometry profiling of MCF-7 cells asynchronous (PBS), under 20h thymidine block (G1/S), and after thymidine washout for 7h (G2). **b)** *BMF* and *FOXM1* transcript levels in asynchronous (PBS) and G2-enriched (thymidine + WO) MCF-7 cells.

Data information: In b values are mean ± S.D. from n=4 independent experiments. * *p*≤0.05 (two-tailed paired t-test).

**Figure S6. Analysis of the DNA methylation and FOXO3 putative roles in *BMF* repression and validation of the CRISPR/Cas9-driven genomic deletions in MCF-7 cells. a)** Bisulphite sequencing of CpG-rich islands near the *BMF* promoter in mitotic neonatal HDFs. **b)** *BMF*, *FOXM1* and *FOXO3* transcript levels in siNeg- and siFOXO3-depleted neonatal HDFs. **c-f)** Genomic DNA was extracted from polyclonal MCF-7 cell cultures established after cell sorting (c-e) or from cells immediately after sorting (f), and used for PCR detection of the wild type (blue asterisk) and mutant (red asterisk) alleles of the (c) CRE#1, (d) CRE#2 and (e-f) CRE#3 CRISPR/Cas9-mediated genomic deletions. **g)** *BUB1B* transcript levels in BUB1B overexpressing MCF-7 cells. **h-i)** Genomic DNA was extracted from MCF-7 and MCF-7 BUB1B overexpressing cells immediately after sorting (h) or from polyclonal cell cultures recovered days after cell sorting (i) and used for PCR detection of the wild type (blue asterisk) and mutant (red asterisk) alleles of the CRE#3 CRISPR/Cas9-mediated genomic deletions.

Data information: In b,g values are mean ± S.D. from n=2 independent experiments.

**Figure S7. Cell fate decision in response taxol plus FOXM1 inhibition and cell unviability upon CRISPR/Cas9-mediated CRE#3 genomic deletion. a)** Individual cell fate profiling of DMSO (n=150) or FDI-6 (n=150) MCF-7 cells treated with 500nM TX. MD, mitotic duration. **b)** Cell fate percentage of the experiments described in a. **c)** Individual cell fate profiling of DMSO (n=150) or FDI-6 (n=150) MDA-MB-231 cells treated with 500nM TX. MD, mitotic duration. **d)** Cell fate percentage of the experiments described in c. **e)** *BMF* transcript levels in FOXM1 overexpressing MDA-MB-231 cells. **f)** *FOXM1* transcript levels in FOXM1 overexpressing MDA-MB-231 cells. **g-h)** Genomic DNA was extracted from polyclonal MDA-MB-231 cell cultures established after cell sorting (g) or from cells immediately after sorting (h), and used for PCR detection of the wild type (blue asterisk) and mutant (red asterisk) alleles of the CRE#3 CRISPR/Cas9-mediated genomic deletions. **i)** *BMF* transcript levels in CRE#3 CRISPR/Cas9-deleted MDA-MB-231 cells immediately after sorting.

Data information: In b, d data are mean ± S.D. of n=3 independent experiments; * *p*≤0.05, **** *p*≤0.0001 (two-tailed χ2). In e, f, i data are mean ± S.D. from n=3 independent experiments; ns *p*>0.05, * *p*≤0.05 (two-tailed paired t-test).

**Table S1.** Correlation of mRNA or protein levels with dose responses of drugs (CTRP CTD2).

**Table S2.** sgRNAs used for CRISPR/Cas9.

**Table S3.** Primers used for genotyping.

**Table S4.** Primers used for RT-qPCR.

**Table S5.** Primers used for bisulphite sequencing.

**Table S6.** Primers used for 4C-sequencing.
