# Supplementary figures and images for: FOXM1 repression increases mitotic death upon antimitotic chemotherapy through BMF upregulation
Source: Cell Death Dis. 2021 May 25;12(6):542. doi: 10.1038/s41419-021-03822-5 (PMC8149823; doi:10.1038/s41419-021-03822-5)

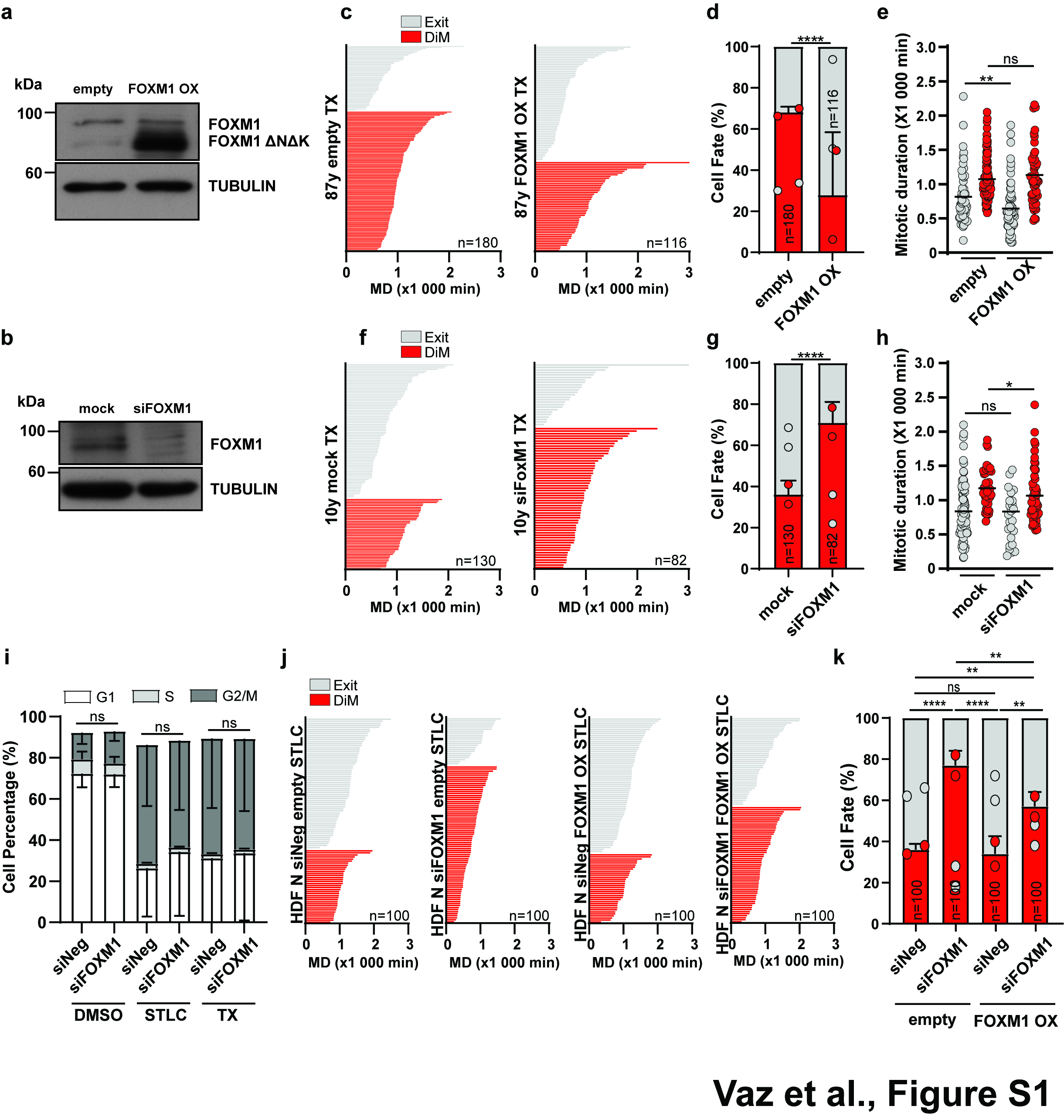

Supplement: Supplementary file 2 — Figure S1 [file 41419_2021_3822_MOESM2_ESM.tif]

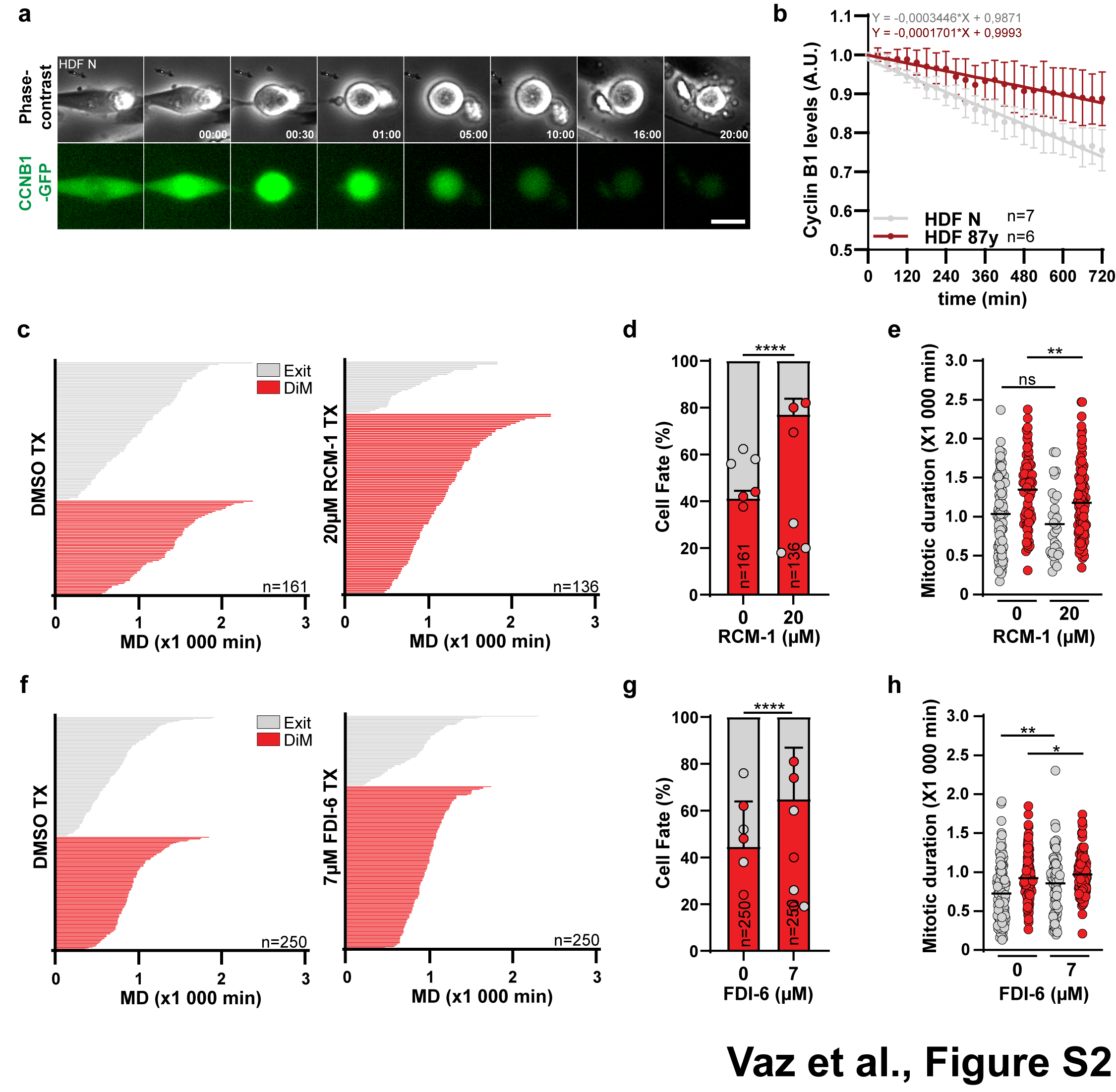

Supplement: Supplementary file 3 — Figure S2 [file 41419_2021_3822_MOESM3_ESM.tif]

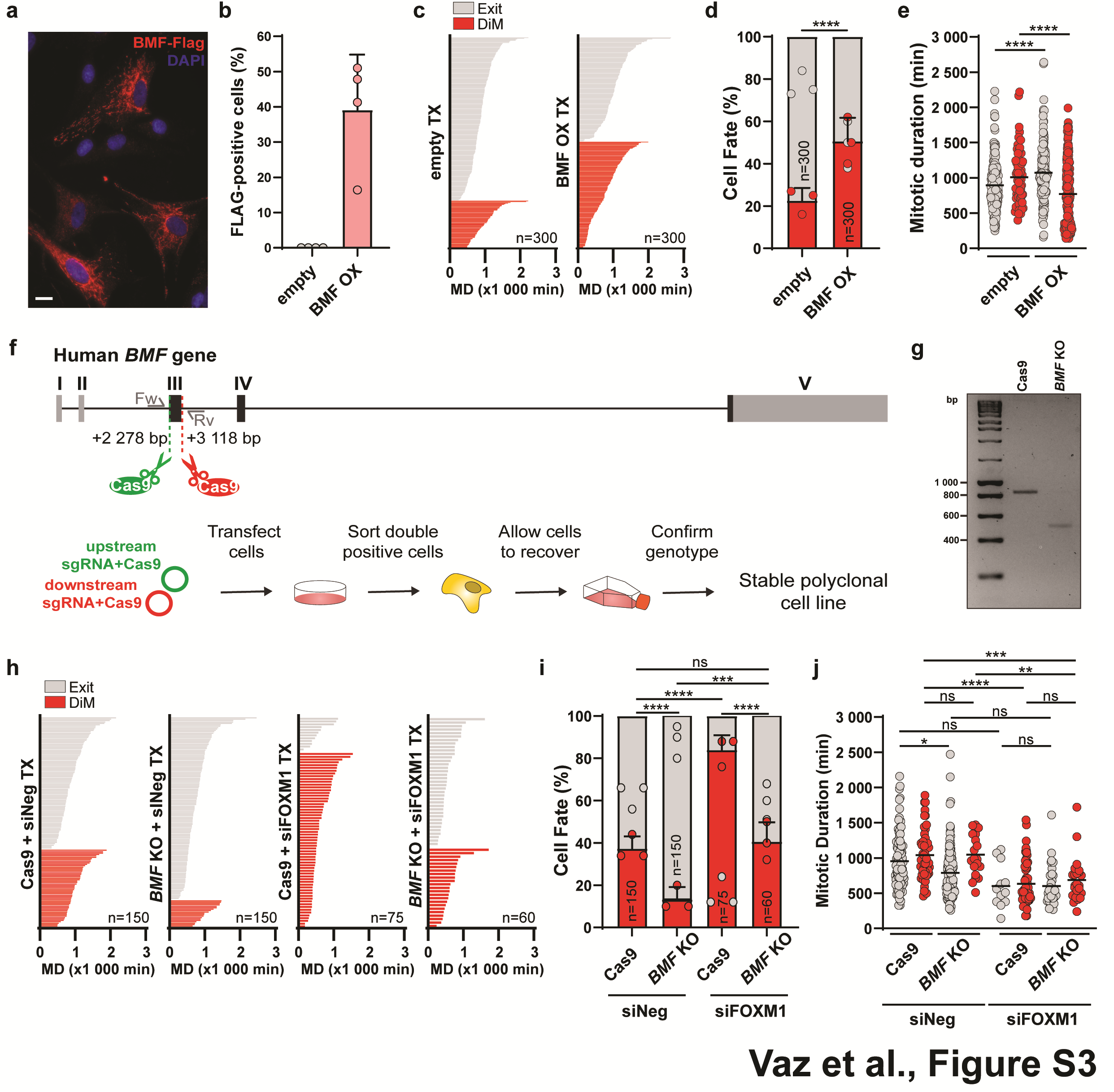

Supplement: Supplementary file 4 — Figure S3 [file 41419_2021_3822_MOESM4_ESM.tif]

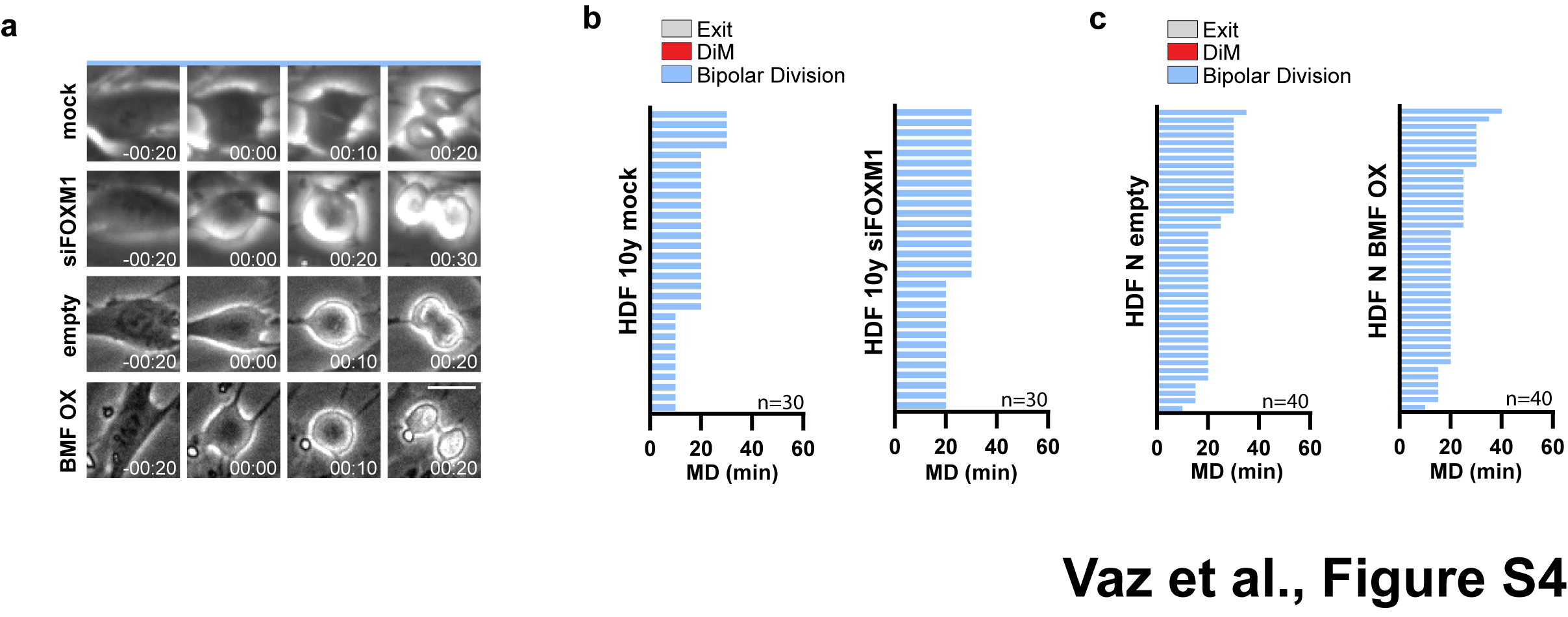

Supplement: Supplementary file 5 — Figure S4 [file 41419_2021_3822_MOESM5_ESM.tif]

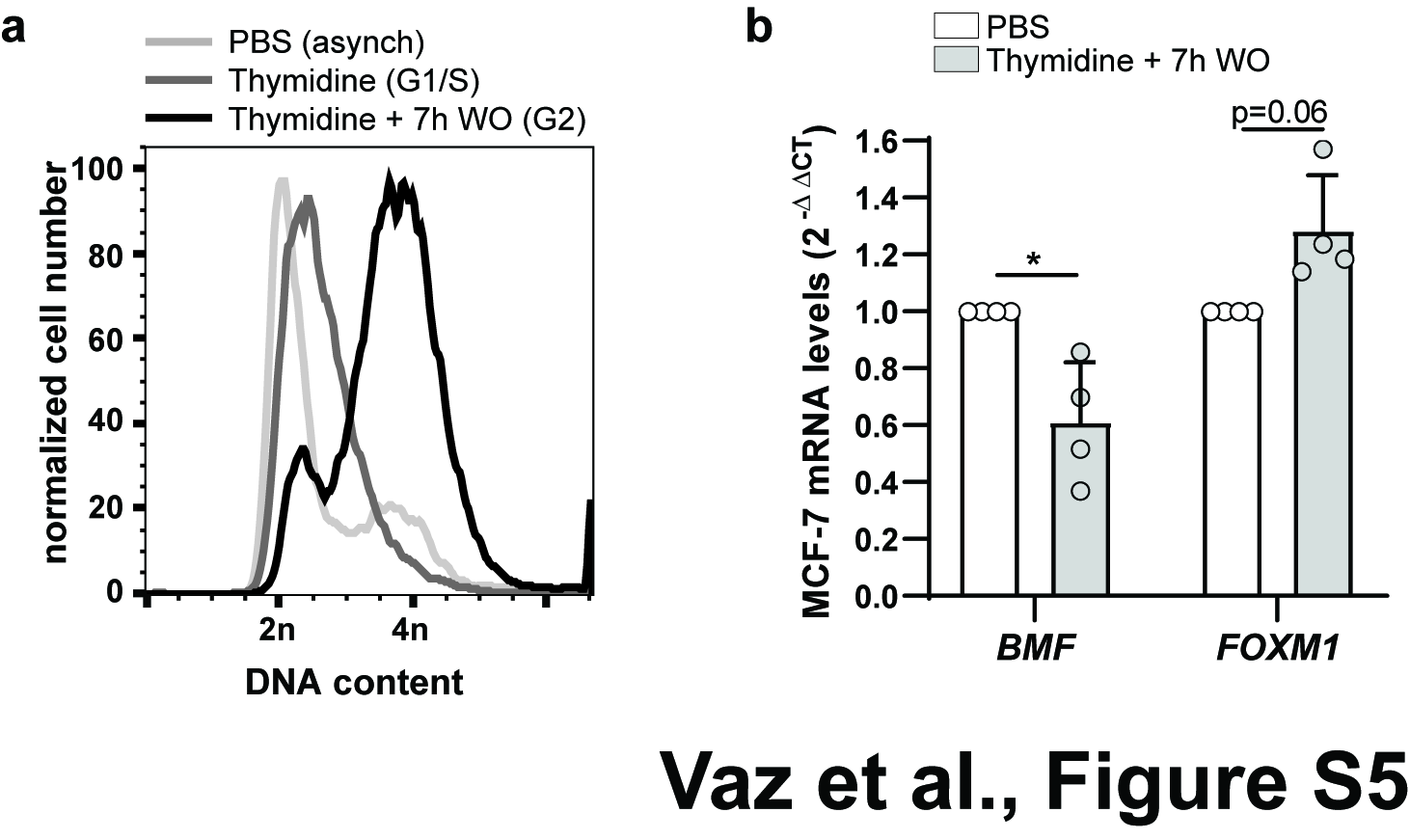

Supplement: Supplementary file 6 — Figure S5 [file 41419_2021_3822_MOESM6_ESM.tif]

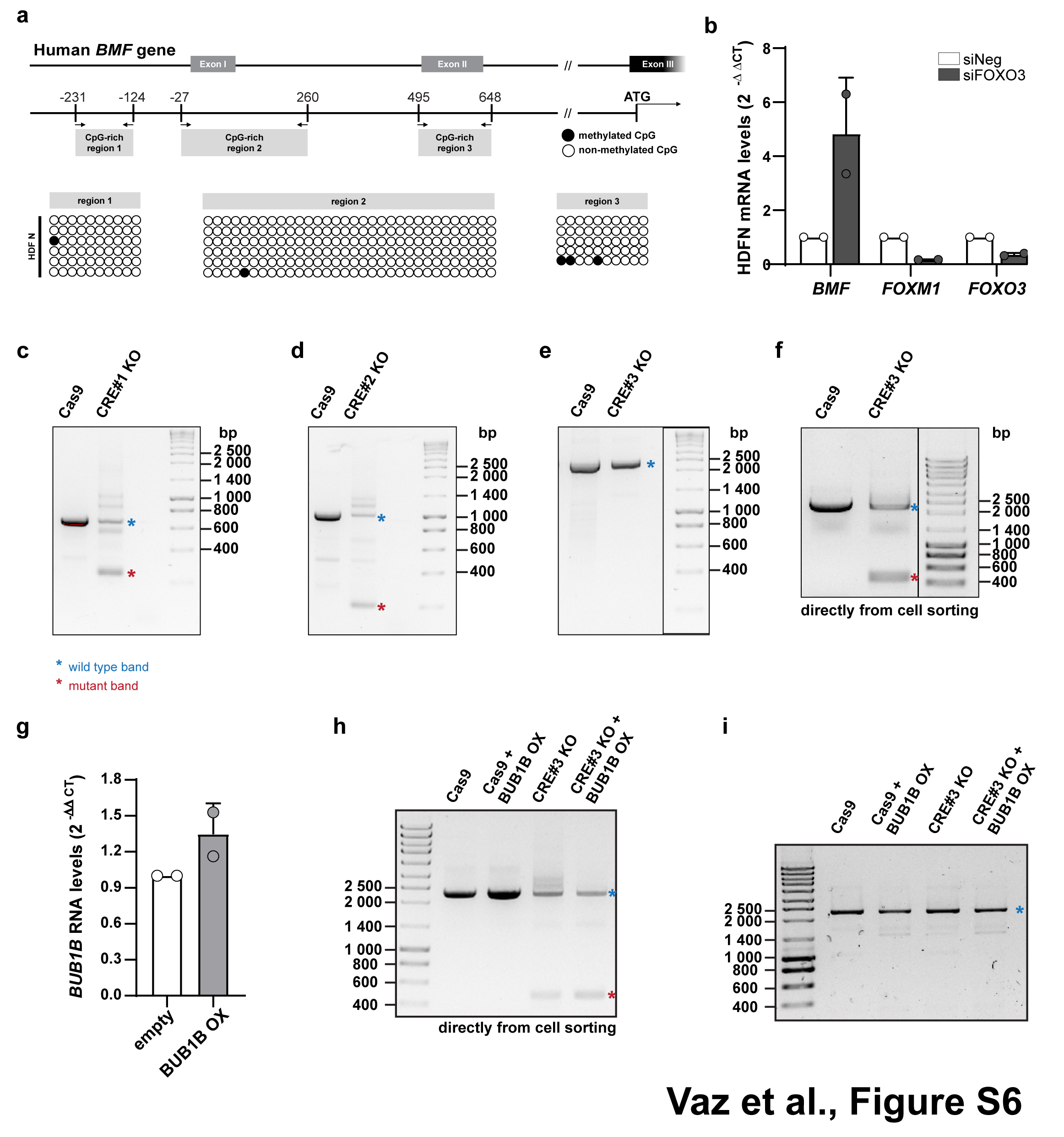

Supplement: Supplementary file 7 — Figure S6 [file 41419_2021_3822_MOESM7_ESM.tif]

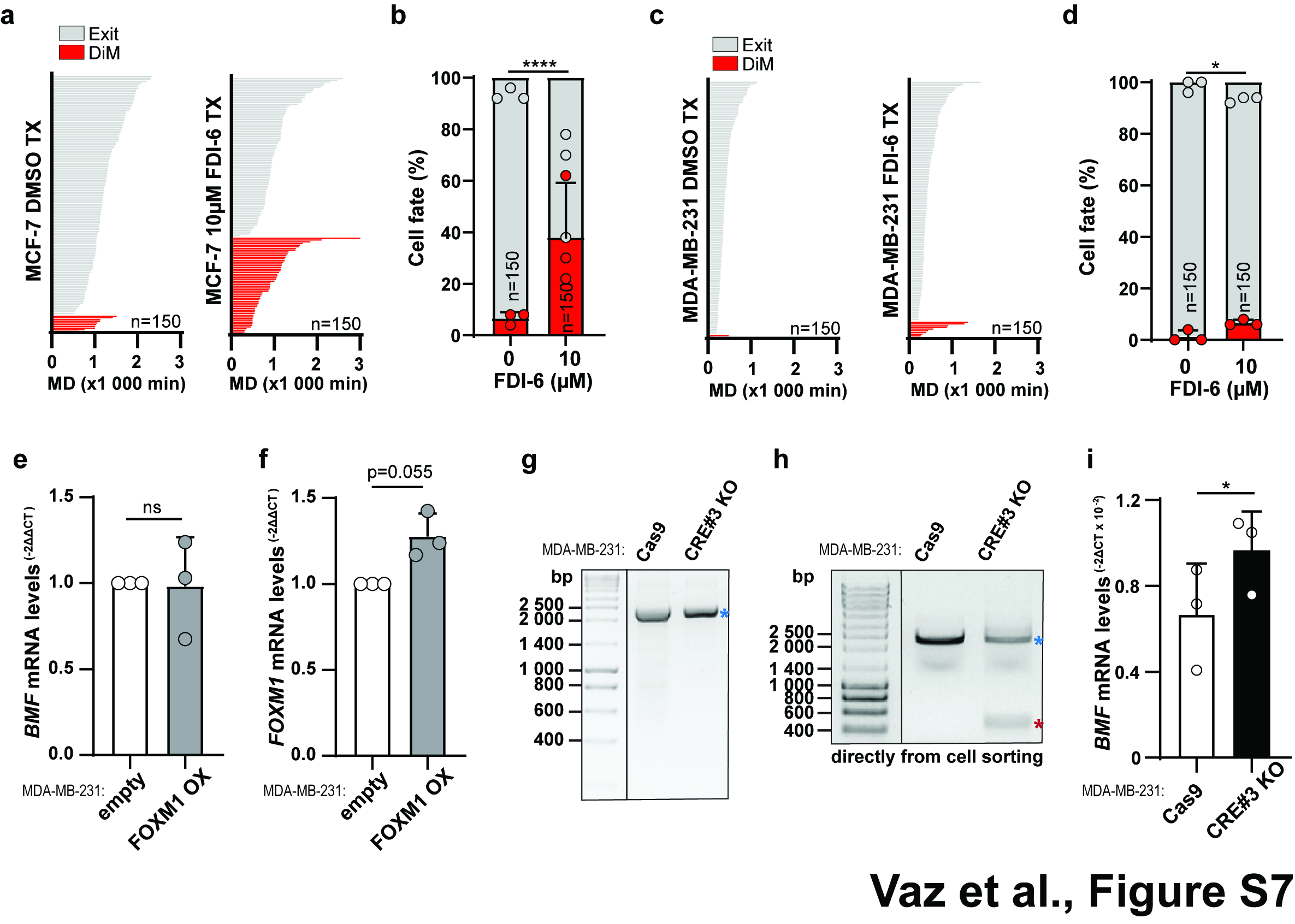

Supplement: Supplementary file 8 — Figure S7 [file 41419_2021_3822_MOESM8_ESM.tif]
