# Supplementary material for: FOXM1 repression increases mitotic death upon antimitotic chemotherapy through BMF upregulation
Source: Cell Death Dis. 2021 May 25;12(6):542. doi: 10.1038/s41419-021-03822-5 (PMC8149823; doi:10.1038/s41419-021-03822-5)
Supplement: Supplementary file 9 — Table S1 [file 41419_2021_3822_MOESM9_ESM.pdf]

Table S1. Correlation of mRNA or protein levels with dose responses of drugs (CTRP CTD2).

Target	Drug	Analysis	Number of points	Pearson	Spearman	Slope	Intercept	p-value (linregress)
BMF	Paclitaxel	mRNA	746	-0,155	-0,155	-3,80E-01	8,12E+00	2,15E-05
		protein	88	-0,278	-0,266	-5,43E-01	6,78E+00	8,79E-03
	Doxorubicin	mRNA	43	-0,508	-0,451	-9,30E-01	6,42E+00	5,99E-04
		Protein	93	-0,36	-0,3	-4,28E-01	7,55E+00	3,97E-04
	Docetaxel	mRNA	348	-0,195	-0,198	-4,77E-01	7,75E+00	2,44E-04
		Protein	42	-0,508	-0,451	-9,30E-01	6,42E+00	5,99E-04
BUB1B	Paclitaxel	mRNA	746	-0,248	-0,265	-1,15E+00	1,36E+01	6,56E-12
		protein	334	-0,303	-0,282	-1,45E+00	7,41E+00	1,64E-08
	Doxorubicin	mRNA	775	-0,23	-0,231	-6,67E-01	1,15E-01	8,68E-11
		Protein	344	-0,298	-0,26	-8,89E-01	8,04E+00	1,81E-08
	Docetaxel	mRNA	348	-0,303	-0,336	-1,30E+00	1,38E+01	7,63E-09
		Protein	155	-0,355	-0,315	-1,66E+00	6,67E+00	5,85E-06
FOXM1	Paclitaxel	mRNA	746	0,041	0,058	1,83E-01	6,10E+00	2,61E-01
		protein	118	-0,022	-0,004	-7,74E-02	7,30E+00	8,17E-01
	Doxorubicin	mRNA	775	0,057	0,065	1,58E-01	6,87E+00	1,12E-01
		Protein	123	-0,069	-0,038	-1,48E-01	7,94E+00	4,59E-01
	Docetaxel	mRNA	348	0,014	0,028	5,85E-02	6,20E+00	7,98E-01
		Protein	56	-0,181	-0,134	-5,44E-01	6,28E+00	1,82E-01
B2M	Paclitaxel	mRNA	746	-0,008	-0,027	-2,07E-02	7,39E+00	8,35E-01
		protein	334	0,054	0,032	1,46E-01	7,49E+00	3,28E-01
	Doxorubicin	mRNA	775	-0,022	-0,023	-3,75E-02	8,19E+00	5,41E-01
		Protein	344	0,042	0,067	6,92E-02	8,08E+00	4,35E-01
	Docetaxel	mRNA	348	-0,052	-0,055	-1,39E-01	8,01E+00	3,31E-01
		Protein	155	0,01	-0,025	2,89E-02	6,72E+00	8,97E-01
