# Supplementary material for: FOXM1 repression increases mitotic death upon antimitotic chemotherapy through BMF upregulation
Source: Cell Death Dis. 2021 May 25;12(6):542. doi: 10.1038/s41419-021-03822-5 (PMC8149823; doi:10.1038/s41419-021-03822-5)
Supplement: Supplementary file 10 — Table S2 [file 41419_2021_3822_MOESM10_ESM.pdf]

Table S2. sgRNAs used for CRISPR/Cas9.

Target	Location	Primer orientation	Primer sequence
BMF KO	upstream target	Fw	caccGCTCCATCTCTCCTGTGAGG
		Rv	aaacCCTCACAGGAGAGATGGAGC
	downstream target	Fw	caccgACTCCTCCCATAGAGACTGG
		Rv	aaacCCAGTCTCTATGGGAGGAGTc
CRE#1 KO	upstream target	Fw	caccGCACTGCAGCTTATGGAGGG
		Rv	aaacCCCTCCATAAGCTGCAGTGC
	downstream target	Fw	caccgTCTGGGTTGAGCAAAGTAAG
		Rv	aaacCTTACTTTGCTCAACCCAGAc
CRE#2 KO	upstream target	Fw	caccgAGACTGTAGCCTCTCCCGAG
		Rv	aaacCTCGGGAGAGGCTACAGTCTc
	downstream target	Fw	caccgTAAATAGCTTGACCATGACT
		Rv	aaacAGTCATGGTCAAGCTATTTAc
CRE#3 KO	upstream target	Fw	caccgCAAGACAAGCCTGACCGACA
		Rv	aaacTGTCGGTCAGGCTTGTCTTGc
	downstream target	Fw	caccgTCTCAATATGCTCTGCAACA
		Rv	aaacTGTTGCAGAGCATATTGAGAc
