# Supplementary material for: FOXM1 repression increases mitotic death upon antimitotic chemotherapy through BMF upregulation
Source: Cell Death Dis. 2021 May 25;12(6):542. doi: 10.1038/s41419-021-03822-5 (PMC8149823; doi:10.1038/s41419-021-03822-5)
Supplement: Supplementary file 14 — Table S6 [file 41419_2021_3822_MOESM14_ESM.pdf]

Table S6. Primers used for 4C-sequencing

Target	Name	Primer sequence
BMF CRE#3	BMF_Read1 (replicate 1)	CCATCTCATCCCTGCGTGTCTCCGACTCAGTTCTAAGAGACGATTCAAAGCAGAAAGTCAGGGATC
	BMF_Read2 (replicate 2)	CCATCTCATCCCTGCGTGTCTCCGACTCAGCTGGCAATCCTCGATTCAAAGCAGAAAGTCAGGGATC
	BMF_Non-read	CCTCTCTATGGGCAGTCGGTGATAACGCAACTGGAAGACACTGG
